# FTO-Eci1 Axis Mediates Exercise-Induced Cardioprotection in Pressure Overload Mice

**DOI:** 10.3390/biom16010098

**Published:** 2026-01-07

**Authors:** Jinyun Wang, Zaoshang Chang, Shuo Lin, Guangyuan Sha, Wenyan Zeng, Qirong Huang, Qibin Deng, Shen Wang, Min Hu, Jingbo Xia

**Affiliations:** 1 Guangdong Provincial Key Laboratory of Physical Activity and Health Promotion, Guangzhou Sport University, Guangzhou 510500, China; 105852023100042@stu.gzsport.edu.cn (J.W.); 105852024100047@stu.gzsport.edu.cn (S.L.); 105852024400273@stu.gzsport.edu.cn (G.S.); 105852025100123@stu.gzsport.edu.cn (W.Z.); 105852025100120@stu.gzsport.edu.cn (Q.H.); 105852025100127@stu.gzsport.edu.cn (Q.D.); wangs@gzsport.edu.cn (S.W.); 2 Department of Physiology, Shaoyang University, Shaoyang 422000, China; 3986@hnsyu.edu.cn

**Keywords:** exercise, myocardial remodeling, FTO, Eci1, N6-methyladenosine

## Abstract

Regular exercise enhances heart function and metabolism. The N6-methyladenosine (m^6^A) RNA modification is related to myocardial homeostasis, with the demethylase fat mass and obesity-associated protein (FTO) crucial for myocardial remodeling. However, its role in exercise-induced heart protection is unclear. We analyzed m^6^A levels and methylation enzymes to evaluate FTO changes in transverse aortic constriction (TAC) mice hearts after six weeks of treadmill exercise. Further in vivo experiments explored the effect of FTO. High-throughput sequencing identified the target gene enoyl-CoA delta isomerase 1 (Eci1). Cardiac-specific *Eci1* knockout mice were used to assess the role of Eci1. The influence of FTO on Eci1 expression was explored by eliminating demethylase activity. The results showed that exercise increased FTO expression in TAC mice hearts. Reducing FTO in the heart diminishes exercise benefits. The differential m^6^A-modified genes in TAC mice hearts were enriched in fatty acid metabolism, with increased methylation of *Eci1* m^6^A and decreased protein levels, leading to abnormal lipid accumulation. Exercise could reverse these effects. *Eci1* knockout partially weakened exercise benefits. FTO regulated Eci1 expression via m^6^A modification, and inhibiting FTO demethylase activity blunted its protective effects on hypertrophic cardiomyocytes. Thus, FTO modulates Eci1 expression through m^6^A-dependent mechanisms, facilitates fatty acid metabolism and mitigates pressure overload-induced heart failure during exercise.

## 1. Introduction

Heart failure is a heterogeneous syndrome that still ranks as a top cause of mortality, despite extensive efforts over the years to develop new treatments [1]. Regular exercise training has been shown to enhance cardiac performance and metabolism [2,3]. The primary consequence of pressure overload is the hypertrophy of cardiomyocytes [4]. Persistent myocardial hypertrophy, resulting from pathological conditions, may eventually lead to heart failure. Although the benefits of exercise have been widely recognized, the underlying mechanisms that initiate and coordinate these benefits remain largely elusive.

Recent studies have identified N6-methyladenosine (m^6^A) RNA modification as essential for maintaining myocardial homeostasis and responding to pathological cardiac processes [5,6,7,8]. The m^6^A modification is a highly conserved and widely distributed modification in mammalian cells, playing a critical role in post-transcriptional gene regulation [9]. As the most abundant internal chemical modification in RNA, m^6^A contributes to the regulation of RNA processing, nuclear export, translation regulation, and RNA degradation [10,11]. The modification of m^6^A is dynamically regulated by m^6^A methyltransferases (METTL3, METTL14, WTAP), m^6^A demethylases (FTO, ALKBH5), and binding proteins (YTHDF1, YTHDF3 and YTHDC2) [12]. The fat mass and obesity-associated protein (FTO), a member of the AlkB protein family, is localized within nuclear speckles where it functions to remove m^6^A residues from RNA [13]. FTO was originally reported as a demethylase for N3-methylthymidine in single-stranded DNA [14] and N3-methyluridine in single-stranded RNA in vitro [15]. It plays a critical role in normal cardiac development, and its dysregulated expression has been implicated in various cardiovascular abnormalities, including hypertrophic cardiomyopathy, arrhythmia, and coronary heart disease [16,17]. Jia et al. demonstrated the involvement of FTO in the reversible modification of m^6^A in mRNA [13]. Subsequently, Mathiyalagan and colleagues reported reduced FTO expression and increased m^6^A levels in both failing mammalian hearts and hypoxic cardiomyocytes. Notably, overexpression of FTO in mice was found to alleviate ischemia-induced myocardial remodeling [7]. Conversely, mice with a cardiomyocyte-specific knockout of the RNA demethylase FTO exhibited compromised cardiac function [18]. Furthermore, numerous studies have investigated the association between FTO and the risk of hypertension, ischemia–reperfusion injury, heart failure, and atherosclerosis [19,20,21,22]. Research on FTO has significantly advanced our understanding of the role of m^6^A methylation in the onset and progression of heart failure. Nevertheless, there remains a paucity of definitive evidence regarding the impact of exercise training on heart function in heart failure patients through the modulation FTO-regulated m^6^A methylation.

Cellular energy metabolism is predominantly maintained by mitochondrial β-oxidation of both saturated and unsaturated fatty acids, with enoyl-CoA delta isomerase 1 (Eci1) acting as a vital coenzyme in the β-oxidation process of unsaturated fatty acids [23]. Under physiological conditions, the myocardium primarily relies on free fatty acids for the generation of high-energy ATP [24]. However, a failing heart demonstrates a diminished ability to metabolize fatty acids [25]. Lipid accumulation in the heart may have a negative impact on the heart and contribute to the progression of heart failure [26]. Clinical studies have also indicated significant lipid deposition in the myocardium of heart failure patients, which is associated with myocardial systolic dysfunction and heart failure [27]. This study aims to elucidate the role of FTO in myocardial remodeling and the development of heart failure through exercise intervention, as well as to investigate the relationship between FTO regulation of cardiac gene expression and function and fatty acid metabolism. Our findings demonstrated that FTO expression was downregulated in failing hearts, while m^6^A levels were upregulated, which was associated with reduced cardiac function and increased lipid accumulation. Exercise intervention was found to enhance cardiac function, reduce lipid accumulation, and restore FTO expression in mice with heart failure. Furthermore, we acquired comprehensive heart transcriptome profiles of altered m^6^A modifications using RNA sequencing and MeRIP-seq techniques in normal, transverse aortic constriction (TAC), and exercise-intervened TAC mice. We subsequently identified and analyzed the biological functions of Eci1, which exhibited differential m^6^A modification and expression. Mechanistically, FTO may regulate fatty acid metabolism mediated by Eci1 in an m^6^A modification-dependent manner, thereby playing a crucial regulatory role in the amelioration of heart failure through exercise.

## 2. Materials and Methods

### 2.1. Animals

Male C57BL/6J mice were purchased from Guangdong Medical Laboratory Animal Center (Guangzhou, China). Eci1-LoxP-targeted (Eci1^fl/fl^) mice (Name: C57BL/6J-Eci1^em1(flox)Cya^, Serial Number: CKOCMP-13177-Eci1-B6J-VA) were created by Cyagen Biosciences (Suzhou, China). The Eci1^fl/fl^ mice were crossed with Myh6-creEsr1 mice to generate Eci1^fl/fl^ Myh6-creEsr1 mice (cKO) with cardiomyocyte specific knockout of Eci1. All animals were genotyped by PCR using specific primers including Eci1^fl/fl^ Primers 1 (F1: 5′-GCAAATTATCTAGTTGCTGCTCCAG-3′, R1: 5′-CCAAAGTAGGTATGGGGAGAACA-3′), Primers 2 for Myh6-creEsr1 (C001009) transgene (F: 5′-TCTATTGCACACAGCAATCCA-3′, R: 5′-CCAGCATTGTGAGAACAAGG-3′). The tissue-specific gene deletion can be confirmed by adding one additional primer (F1: 5′-GCAAATTATCTAGTTGCTGCTCCAG-3′, R2: 5′-GATGGCTTCCCTCCTTCTGTAAG-3′). The selective estrogen receptor modulator tamoxifen is widely used in preclinical experiments to induce conditional gene deletion in mice using the Cre-loxP recombination method [28]. The administration of tamoxifen was carried out by intraperitoneal injection, using tamoxifen (10540-29-1, Sigma, Santa Ana, CA, USA) dissolved in corn oil (8001-30-7, Solarbio, Beijing, China) at 10 mg/mL. Male Eci1-cKO (seven-week-old) mice were administered tamoxifen at a dosage of 25 mg/kg/day for five consecutive days, followed by a one-week rest period prior to subsequent experimentation. Male age-matched Eci1^fl/fl^ mice lacking Myh6-creEsr1 served as control subjects and received an equivalent dosage of tamoxifen. All mice were maintained under specific-pathogen-free conditions with ad libitum access to water and food. The light cycle was maintained at 12 h light/12 h dark. All animal experiments were conducted in accordance with Institutional Animal Care Guidelines and with the approval of the Animal Experimental Ethics Inspection of Guangzhou Sport University (2022DWLL-10).

### 2.2. Grouping of Animals

In the investigation of exercise intervention as an intervention to ameliorate TAC-induced heart failure, eight-week-old male C57BL/6J mice of the 19–23 g weight range were used. The animal subjects were allocated into three groups: Sham, TAC, and TAC exercise (TAC-Ex), with each group comprising eight mice. Among all groups of animals, the body weight range was similar (225–250 g). For protein analysis, three samples were randomly selected. For quantitative real-time PCR (qRT-PCR) analysis, six samples were randomly chosen.

In the in vivo experiment involving FTO inhibition, eight-week-old male C57BL/6J mice of the 19–23 g weight range were used. The animals were classified into five groups: Sham, TAC, TAC-Ex, TAC-Ex+AAV-NC, and TAC-Ex+AAV-FTO, each consisting of eight mice.

In the *Eci1* cardiac-specific knockout mouse experiment, seven-week-old male mice of the 18–22 g weight range were used. All genotypes were tamoxifen treated. The mice were divided into five groups: Sham, TAC-flox, TAC-cKO, TAC-Ex-flox, and TAC-Ex-cKO, with eight mice per group. For protein analysis in this experiment, three samples were randomly selected.

### 2.3. Transverse Aortic Constriction Surgery

TAC was performed as previously described [29]. Briefly, eight-week-old male mice were anesthetized using isoflurane and ventilated with a mixture of 1 L/min oxygen and 1.5% isoflurane. After positioning the mice appropriately, a thoracotomy was performed under a microscope to expose the thymus. The thymus and surrounding adipose tissue were carefully dissected from the aortic arch. Subsequently, the aortic arch was ligated with a 7-0 silk suture against a 27-gauge needle, which was promptly removed following ligation. In sham-operated mice, the procedure was identical except for the exclusion of the aortic constriction step. Post-surgery, mice were continuously monitored until full recovery.

### 2.4. Adeno-Associated Virus Injection

We employed adeno-associated virus serotype 9 (AAV9) vectors to downregulate FTO expression in cardiac tissue. The AAV9 vectors were packaged and produced by BrainVTA Co., Ltd. (Wuhan, China). For in vivo delivery of AAV9-cTNT-EGFP-shRNA-FTO, mice received three tail vein injections of 100 μL AAV at a titer of 1 × 10^12^ v.g./mL. AAV9-cTNT-EGFP-shRNA-NC, containing nonsense sequences, was used as a control [30]. The shRNA target sequence for FTO was 5′-GCACCTTGGATTATATCTTAG-3′.

### 2.5. Treadmill Exercise Protocol

Treadmill exercise intervention commenced one-week post-TAC surgery, occurring five times per week over an eight-week period. The exercise protocol was used according to the reported literature with some modifications [31]. The exercise intervention phase for 6 weeks was set at a speed of 12 m/min, 1 h/day, 5 days/week, and slope = 0°. The exercise intervention was performed at 19:00 pm. Animals were allowed to break three times for 3–5 min during each session.

### 2.6. Echocardiographic Assessment

Echocardiography was employed to noninvasively evaluate cardiac function, following a previously established methodology [32]. In brief, mice were anesthetized using 1.5% to 2% isoflurane and assessed using a high-resolution ultrasound imaging system (VINNO 6, Vinno Corporation, Suzhou, China) in M-mode with a 23 MHz probe. The inhalation flow was adjusted to maintain the heart rate at 400–500 beats/min. Cardiac function was assessed by calculating the ejection fraction (EF) and fractional shortening (FS) from M-mode images.

### 2.7. Primary Cardiomyocytes Isolation and Treatment

Neonatal cardiomyocytes were isolated and cultured from the ventricular tissue of 1- or 2-day-old mice, in accordance with established protocols [33]. To simulate cardiomyocyte hypertrophy in vitro, the cell model was incubated with angiotensin II (Ang II, HY-13948, MCE, Shanghai, China) for 48 h. The CCK8 assay (C0042, Beyotime, Shanghai, China) and detection of hypertrophic phenotype were performed at varying concentrations (0, 2, 6, 10, 14 μM) to determine the optimal concentration.

### 2.8. Lentiviral Transduction in Cardiomyocytes

Gene silencing and overexpression in primary cardiomyocytes were achieved by lentiviral vector expression system according to the established methods [34]. To investigate the role of Eci1 in cardiomyocyte hypertrophy, we developed Eci1 expression and shRNA-Eci1 expression plasmids. Additionally, to explore the mechanism by which FTO regulates Eci1 expression in an m^6^A-dependent manner, we constructed plasmids for both wild-type FTO and the catalytic mutant FTO^R96Q^ (FTO-mut) [20]. The vectors utilized are detailed in Table 1.

### 2.9. Histology and Immunofluorescent Staining

Six weeks after the exercise intervention, mice were anesthetized with isoflurane, and blood samples were collected via orbital extraction to obtain serum. Subsequently, the mice were euthanized through cervical dislocation, and their hearts were promptly excised. To assess myocardial hypertrophy progression, we determined the heart weight to body weight (HW/BW) and heart weight to tibia length (HW/TL) ratios. The excised hearts were fixed in 4% paraformaldehyde, embedded in paraffin, and sectioned into 5 μm slices. These sections were subjected to staining with Masson’s trichrome kit (G1006, Servicebio, Wuhan, China), hematoxylin and eosin kit (H&E) (G1005, Servicebio, Wuhan, China), Oil Red O kit (G1015, Servicebio, Wuhan, China), and wheat germ agglutinin (WGA) (W11261, Thermo Fisher, MA, USA) for comprehensive analysis. To analyze lipid droplets in cardiomyocytes, Oil Red O staining was conducted according to the product instruction (G1015, Servicebio, Wuhan, China). In the immunofluorescence experiments, cardiomyocytes were stained following previously established protocols [35]. Initially, cardiomyocytes underwent fixation, permeabilization, and blocking, followed by incubation with primary antibodies. Post-washing with PBS, the sections were treated with appropriate secondary antibodies and subsequently counterstained with DAPI (Sigma, Sigma-Aldrich, St. Louis, MO, USA). The primary antibodies utilized included anti-Ki67 (1:200, ab15580, Abcam, Cambridge, UK) and anti-cTnT (1:100, sc-20025, Santa Cruz, CA, USA). The secondary antibodies employed were DyLight 594 conjugated affiniPure goat anti-rabbit IgG (1:200, BA1142, BOSTER, Wuhan, China) and Alexa Fluor 647 AffiniPure goat anti-mouse IgG (1:200, 115-605-003, Jackson ImmunoLabs, West Grove, PA, USA). The fibrosis area was quantified using the Pannoramic Scanner system (Pannoramic 250, 3D HISTECH, Budapest, Hungary). Imaging of the WGA, Oil Red O, and immunofluorescence staining was conducted using a fluorescence microscope (DM4B, Leica, Wetzlar, Germany) or a Zeiss LSM 700 laser confocal microscope (Carl Zeiss, Oberkochen, Germany).

### 2.10. Free Fatty Acid (FFA), Triglyceride (TG) and ATP Measurement

Concentrations of FFA and TG in plasma, myocardial tissues and cultured cardiomyocytes were measured with a Free Fatty Acid Assay Kit (A042-2-1, Nanjing Jiancheng Bioengineering Institute, Nanjing, China) and Triglyceride Assay Kit (A110-1-1, Nanjing Jiancheng Bioengineering Institute, Nanjing, China), respectively, in accordance with the manufacturer’s instructions. The ATP content in myocardial tissues was assessed using an ATP bioluminescent assay kit (MAK473-1KT, Sigma, Sigma-Aldrich, St. Louis, MO, USA).

### 2.11. Apoptosis Measurement by Flow Cytometry

In order to assess apoptosis in cardiomyocytes, a commercial kit (KGA1016, KeyGEN Biotech, Nanjing, China) utilizing Annexin V-PE/7-AAD dual staining was employed to quantify apoptotic cell rates, following the manufacturer’s instructions. In summary, cells were harvested, washed with PBS, resuspended in binding buffer, and incubated in the dark at room temperature for 10 min with a mixture containing 1 μL of Annexin V-PE and 5 μL of 7-AAD. The results were analyzed within one hour using a flow cytometer (CytoFLEX LX, BECKMAN, Brea, CA, USA).

### 2.12. Quantification of Total m^6^A Levels

The m^6^A levels of total RNA were detected via a colorimetric ELISA assay using the EpiQuik m^6^A RNA Methylation Quantification Kit (P-9005-48, Epigentek, Farmingdale, NY, USA), in accordance with the manufacturer’s instructions. Quantification of m^6^A levels was performed with a microplate reader (MQX200, BioTek, Winooski, VT, USA).

### 2.13. MeRIP-Seq and RNA-Seq

Following six weeks of TAC surgery, three samples from the Sham, TAC, and TAC-Exercise (TAC-Ex) groups were randomly selected for sequencing. Library preparation and high-throughput sequencing were carried out by SHBIO Biotechnology Corporation in Shanghai, China. Utilizing the sequencing data, KEGG pathway enrichment analysis was performed on differentially methylated genes, focusing on energy metabolism pathways. This was followed by an analysis of transcriptional expression of genes within significantly enriched fatty acid metabolism pathways. The objective was to identify target genes that demonstrate significant upregulation or downregulation in the TAC model group, with a notable reversal effect observed following exercise intervention.

### 2.14. MeRIP-qPCR

Total RNA was extracted using the RNeasy Kit (RC112, Vazyme Biotech, Nanjing, China). Following the protocol for the inactivation of methylated RNA immunoprecipitation (MeRIP) m^6^A kit (Bersinbio, Guangzhou, China), the RNAs were incubated with an anti-m^6^A antibody (ab284130, Abcam, Cambridge, UK) for immunoprecipitation. The m^6^A-RIP-qPCR was performed according to a previously established protocol [36].

### 2.15. RIP Assay

The RNA immunoprecipitation (RIP) assay was conducted as previously described [37]. In brief, cardiomyocytes were harvested post-Ang II treatment and lysed using RIP lysis buffer (Millipore, Billerica, MA, USA). Subsequently, 2 μg of anti-FTO antibody and normal rabbit IgG were incubated with Magnetic Beads Protein A/G for 1 h before being mixed with the cell lysate overnight at 4 °C. The pull-down complex was digested with proteinase K, and RNA was extracted for quantitative reverse transcription PCR (qRT-PCR).

### 2.16. Western Blot Analysis

Mouse heart tissues or cultured cardiomyocytes were lysed utilizing RIPA lysis buffer (P0013B, Beyotime, Shanghai, China). Proteins intended for Western blot analysis were quantified accordingly. The primary antibodies employed included: anti-Bax (50599-2-Ig, Proteintech, Wuhan, China), anti-Bcl-2 (68103-1-Ig, Proteintech, Wuhan, China), anti-Cleaved Caspase-3 (ab214430, Abcam, Cambridge, UK), anti-Caspase-3 (19677-1-AP, Proteintech, Wuhan, China), anti-Eci1 (11535-1-AP, Proteintech, Wuhan, China), anti-FTO (GB111359-100, Servicebio, Wuhan, China), and anti-β-actin (66009-1-Ig, Proteintech, Wuhan, China). Secondary antibodies used were HRP-conjugated Goat Anti-Mouse IgG (SA00001-1, Proteintech, Wuhan, China) and HRP-conjugated Goat Anti-Rabbit IgG (SA00001-2, Proteintech, Wuhan, China). Quantification of the chemiluminescent signal was conducted using Image-Pro Plus software, version 6.0 (Media Cybernetics, Rockville, MD, USA). The relative expression levels of the target protein, normalized to β-actin, were set to one in the control group. Original western blots can be found at Supplementary Materials.

### 2.17. RNA Extraction and qRT-PCR

Total RNA was extracted using the RNeasy Kit (RC112, Vazyme Biotech, Nanjing, China). Complementary DNA (cDNA) was synthesized via reverse transcription using the RT SuperMix Kit (G3337, Servicebio, Wuhan, China). qRT-PCR was performed using the HiScript II One Step qRT-PCR SYBR Green Kit (Q221, Vazyme Biotech, Nanjing, China) on the CFX96 Touch qRT-PCR System (Bio-Rad, CA, USA). GAPDH served as the internal reference control for normalizing gene expression, employing the 2^−ΔΔCt^ method. The specific primer sequences utilized in the qRT-PCR analysis included those for ANP (F: 5′-CGGCTTCCTGCCTTCATCTATCAC-3′, R: 5′-GCGTCTGTCCTTGGTGCTGAAG-3′), BNP (F: 5′-GAGTCCTTCGGTCTCAAGGC-3′, R: 5′-CAACTTCAGTGCGTTACAGC-3′), MYH7 (F: 5′-CAACCTGTCCAAGTTCCGCA-3′, R: 5′-TACTCCTCATTCAGGCCCTTG-3′), FTO (F: 5′-TTCATGCTGGATGACCTCAATG-3′, R: 5′-GCCAACTGACAGCGTTCTAAG-3′), ALKBH5 (F: 5′-CGCGGTCATCAACGACTACC-3′, R: 5′-ATGGGCTTGAACTGGAACTTG-3′), METTL3 (F: 5′-CTGGGCACTTGGATTTAAGGAA-3′, R: 5′-TGAGAGGTGGTGTAGCAACTT-3′), METTL14 (F: 5′-CTCCAAACTCAAAACGGAAGTGT-3′, R: 5′-ATGGGGATTTAAGCTCTGCGT-3′), WTAP (F: 5′-GGCGAAGTGTCGAATGCT-3′, R: 5′-CCAACTGCTGGCGTGTCT-3′), Eci1 (F: 5′-CCTCCCGTGAATTCCCTCAG-3′, R: 5′-CGGCCATACATCTCCAGCAA-3′), GAPDH (F: 5′-GCCCATCACCATCTTCCAGGAGCG-3′, R: 5′-GCAGAAGGGGCGGAGATGATGACC-3′).

### 2.18. Statistical Analysis

Data analysis was executed using GraphPad Prism software, version 9.5.1 (San Diego, CA, USA), with results presented as the mean ± SEM. The number of samples (*n*) for each experiment or group is detailed in the respective figure legends. Prior to statistical analysis, the normality of data distribution was assessed using the Shapiro–Wilk test. Statistical comparisons between two groups were conducted using an unpaired, two-tailed Student’s *t*-test. For analyses involving three or more groups, one-way ANOVA followed by Tukey’s multiple comparisons test was employed. For nonnormality data, Kruskal–Wallis one-way ANOVA was used. A *p*-value of less than 0.05 was considered indicative of statistical significance.

## 3. Results

### 3.1. Exercise Intervention Attenuates TAC Induced Heart Failure and Inhibits the m^6^A Methylation by Increasing FTO Expression

**Figure 1:**
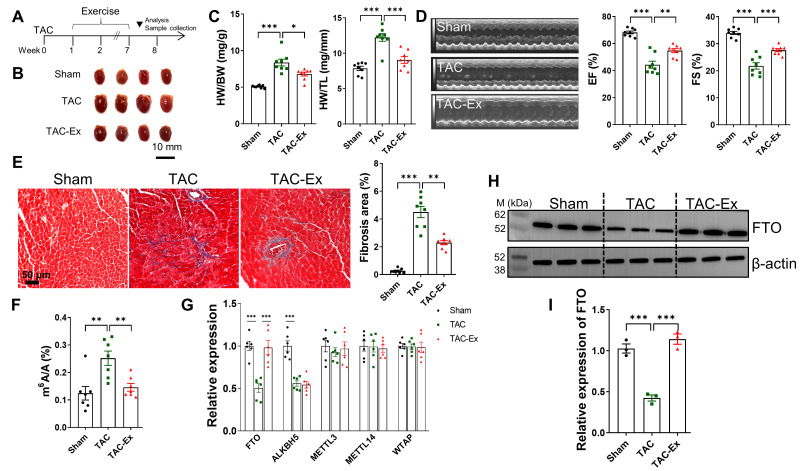
Exercise induced FTO expression during the intervention. (**A**) Schematic diagram of exercise intervention. (**B**) Representative hearts were photographed. Scale bar, 10 mm. (**C**) HW/BW and HW/TL for the indicated groups (*n* = 8 per group). BW, body weight; HW, heart weight; TL, tibia length. (**D**) The evaluation of cardiac function was detected by echocardiography. The EF and FS were then both calculated (*n* = 8 per group). EF, ejection fraction; FS, fractional shortening. (**E**) Representative Masson’s trichrome staining pictures and quantitative results of fibrosis (*n* = 8 per group). Scale bar, 50 µm. (**F**) Analysis of m^6^A levels in myocardial tissues (*n* = 7 per group). (**G**) Expression of methyltransferases (METTL3, METTL14, and WTAP) and demethylases (FTO and ALKBH5) were determined by qRT-PCR (*n* = 6 per group). (**H**) FTO expression across mouse myocardial tissues in three groups (*n* = 3 per group). (**I**) Fold change in FTO expression. Statistical significance is denoted by: * *p* < 0.05, ** *p* < 0.01, *** *p* < 0.001.

TAC resulted in an elevated cardiac hemodynamic load, leading to myocardial remodeling and dysfunction, as previously reported [38]. The timeline for model establishment and exercise intervention is depicted in Figure 1A. Exercise significantly mitigated pathological heart enlargement in TAC mice, as shown in Figure 1B. The HW to BW ratios increased following TAC, along with an increase in HW to TL ratios (Figure 1C), indicating TAC-induced pathological myocardial hypertrophy. However, in TAC mice subjected to exercise, both HW/BW and HW/TL ratios significantly decreased (Figure 1C). Echocardiographic analysis revealed a significant decline in cardiac function in TAC mice compared to the Sham group, as evidenced by reduced EF and FS, which were ameliorated by exercise (Figure 1D). Furthermore, exercise significantly attenuated TAC-induced cardiac fibrosis, as illustrated in Figure 1E. It has been demonstrated that m^6^A methylation is crucial for the development of cardiomyocyte hypertrophy and the maintenance of cardiac homeostasis in adult mice [6]. Subsequently, a quantitative m^6^A assay utilizing ELISA was employed to assess the role of m^6^A modification levels. The global mRNA m^6^A levels in the cardiac tissue of a TAC-induced heart failure model were quantified, a significant elevation was observed when compared with the sham group. Notably, exercise intervention substantially decreased mRNA m^6^A levels in myocardial tissue relative to the TAC group (Figure 1F). To elucidate the expression patterns of m^6^A methyltransferases (METTL3, METTL14, and WTAP) and demethylases (ALKBH5 and FTO), myocardial samples were analyzed using qRT-PCR. Among these five genes, TAC resulted in a marked suppression of *FTO* expression compared with controls, whereas exercise significantly upregulated *FTO* expression, as illustrated in Figure 1G. Consistent with the qRT-PCR findings, increased FTO expression in the TAC-induced heart failure model and its reduction by exercise were also observed at the protein level (Figure 1H,I). These findings imply that FTO-mediated m^6^A modification may be pivotal in attenuating TAC-induced heart failure.

### 3.2. Inhibition of FTO Expression Partially Counteracts the Effect of Exercise on Ameliorating Heart Failure

**Figure 2:**
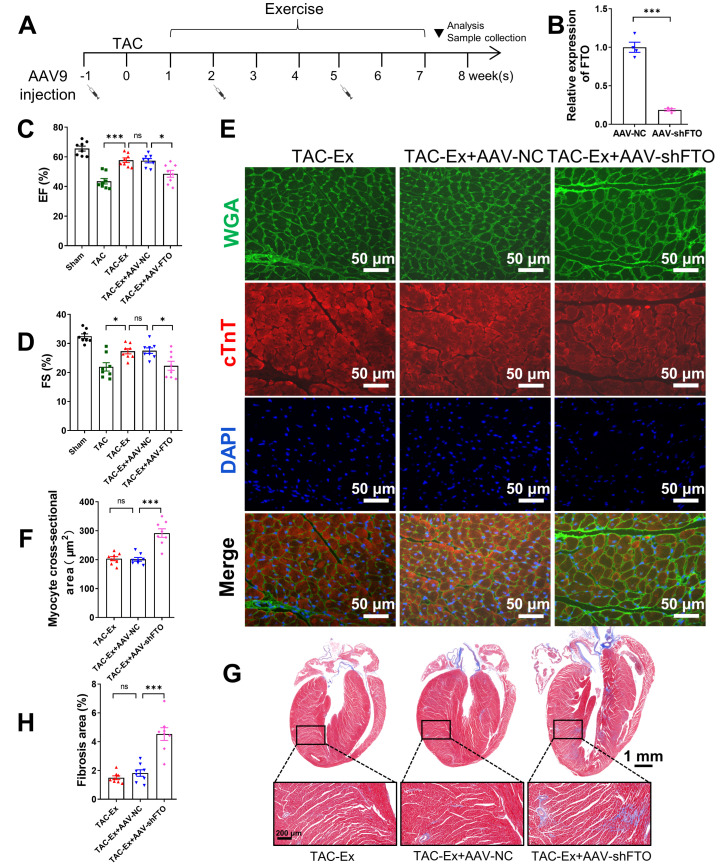
The role of FTO in attenuating heart failure through exercise. (**A**) Schematic diagram of exercise intervention and AAV9 administration. (**B**) Validation of AAV9 knockdown efficiency in vivo (*n* = 4 per group). (**C**) Left ventricular EF. EF, ejection fraction (*n* = 8 per group). (**D**) Left ventricular FS (*n* = 8 per group). FS, fractional shortening. (**E**) Representative WGA staining revealed cardiomyocyte cross sectional area. Wheat germ agglutinin (WGA, green), Cardiac troponin T (cTnT) (red), DAPI (blue). Scale bar, 50 µm. (**F**) Quantification results for WGA staining of heart sections (*n* = 8 per group). (**G**) Representative sections of Masson’s trichrome staining. Macroscopic scale bar, 1 mm; microscopic scale bar, 200 µm. (**H**) Quantitative results of Masson’s trichrome staining (*n* = 8 per group). Statistical significance is denoted by: * *p* < 0.05, *** *p* < 0.001, ns indicates *p* > 0.05.

To investigate the relationship between FTO and exercise intervention, we conducted an exercise intervention while inhibiting FTO expression to demonstrate the association between exercise benefits and FTO. All animals in these groups were from a separate batch, not from previous experiments, and underwent the exercise protocol as described in the Materials and Methods section. We employed AAV9-mediated delivery of shRNA to suppress FTO expression in myocardial tissue, administering a total of three injections over an eight-week period (Figure 2A). Negative control-shRNA (shNC) was used as a control, which was also subjected to the exercise protocol. The successful knockdown of FTO in heart tissue was validated through qPCR analysis of FTO mRNA expression levels (Figure 2B). In comparison with the TAC-Ex+AAV-shNC mice, the TAC-Ex+AAV-shFTO mice exhibited a reduced EF and FS (Figure 2C,D). Histological analysis revealed an increase in cardiomyocyte size in the hearts of TAC-Ex+AAV-shFTO mice compared with TAC-Ex+AAV-shNC mice (Figure 2E,F). Additionally, the TAC-Ex+AAV-shFTO mice demonstrated increased myocardial fibrosis, as indicated by Masson’s trichrome staining (Figure 2G,H). Collectively, these findings suggest that FTO plays a critical role in mitigating heart failure through exercise.

### 3.3. Bioinformatics Analysis and Screening of Target Gene Eci1

**Figure 3:**
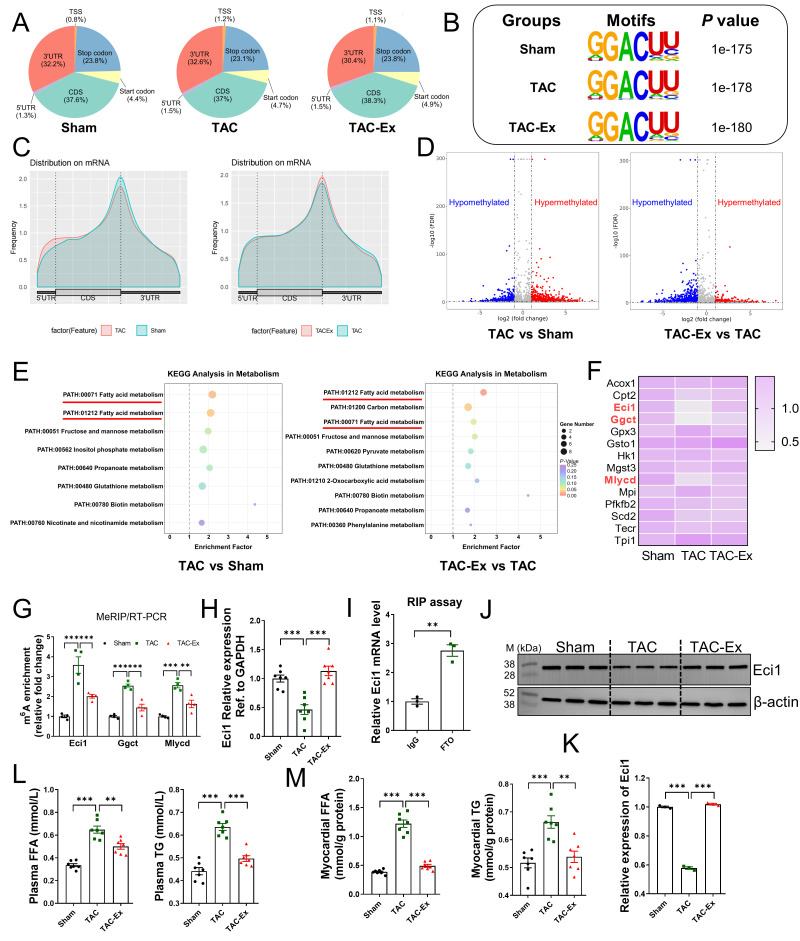
Screening of the candidate genes based on MeRIP-seq and RNA-seq. (**A**) The proportion analysis for specific m^6^A binding sites in MeRIP-seq in three groups. (**B**) Top sequence motif identified from MeRIP-seq peaks in three groups. (**C**) Peak distribution of m^6^A modification in meRIP-seq results between the two compared groups. (**D**) Volcano plot of MeRIP-Seq showed the changed m^6^A peaks between two compared groups. Genes with a 2-fold differentially m^6^A-modified genes and FDR ≤ 0.01 were highlighted in red for hyper-methylation and blue for hypo-methylation. (**E**) KEGG pathway enrichment analysis of MeRIP-seq data. (**F**) Expression heatmap of all genes in the dataset under fatty acid metabolism pathway. Heatmap of the data from RNA-seq. Genes with significant changes in expression (*Eci1*, *Ggct* and *Mlycd*) were labeled in red. (**G**) MeRIP-qPCR assay confirmed the results from MeRIP-seq (*n* = 4 per group). (**H**) *Eci1* mRNA expression was performed using qRT-PCR (*n* = 6 per group). (**I**) RIP assay was performed using IgG or FTO antibody. The enrichment of Eci1 mRNA was measured by qRT-PCR assay (*n* = 3 per group). (**J**) Eci1 protein expression was performed using Western blot. (**K**) Fold change in Eci1 protein expression (*n* = 3 per group). (**L**) FFA levels and TG content in plasma (*n* = 7 per group). (**M**) FFA levels and TG content in myocardial tissues (*n* = 7 per group). FFA, free fatty acid; TG, triglyceride. Statistical significance is denoted by: ** *p* < 0.01, *** *p* < 0.001.

We performed a comprehensive transcriptome-wide analysis of m^6^A RNA methylation employing MeRIP-seq and RNA-seq. Our findings indicated an enrichment of m^6^A peaks within the 5′-untranslated regions, coding sequences, and 3′-untranslated regions (Figure 3A). The robustness of transcriptome data was confirmed by an unbiased motif search, which utilized the identified m^6^A peaks as input and successfully identified the previously reported m^6^A consensus sequence, DRACH (Figure 3B). Quantitative analysis of m^6^A peaks across all transcripts revealed a distribution pattern with a notable enrichment of m^6^A near the translation termination site (Figure 3C). Additionally, differential m^6^A methylation peaks and their statistical significance were assessed using MeTDiff software [39]. The MeRIP-seq analysis identified both hypomethylated and hypermethylated m^6^A peaks between the two comparative groups (Figure 3D). Prior studies have established that energy metabolism disorders are primary risk factors for the onset of cardiovascular diseases, leading to cardiac dysfunction [40,41]. Subsequently, we conducted a KEGG pathway analysis on the differentially methylated transcripts to explore their involvement in metabolic pathways. Among the metabolic pathways analyzed, those related to fatty acid metabolism displayed distinct differences between the two comparative groups (Figure 3E). Figure 3F presents a heatmap of the expression levels of all genes identified within the fatty acid metabolism pathway, encompassing a total of 14 genes. These expression levels, quantified as FPKM values, were obtained from RNA-seq data. Notably, the genes *Eci1*, *Ggct*, and *Mlycd* were significantly downregulated and hyper-methylated in the TAC group, while being upregulated and hypo-methylated in the TAC-Ex group. These observations from MeRIP-seq data were validated by MeRIP-qPCR assays, as shown in Figure 3G. Based on relative enrichment, *Eci1*, which exhibited the highest differential fold change, was selected for further analysis. Furthermore, the expression of Eci1 across the three groups was confirmed using qRT-PCR and Western blot assays (Figure 3H,J,K). Our results indicate that TAC treatment significantly decreased the production of Eci1 protein, whereas exercise intervention resulted in an elevation of Eci1 protein levels (Figure 3J). Additionally, RIP analysis revealed that FTO can bind to *Eci1* mRNA (Figure 3I), suggesting its role as the m^6^A demethylase for *Eci1*. *Eci1* is crucial in mitochondrial function by facilitating β-oxidation through the catalysis of unsaturated fatty acids. Subsequent observations revealed a significant increase in the levels of FFA and TG in both plasma and myocardial tissues within the TAC-induced heart failure model group. Notably, these alterations were reversed following the exercise intervention (Figure 3L,M). These findings suggest an association between abnormal lipid metabolism and Eci1. *Eci1* may serve as a potential target for FTO, warranting further investigation.

### 3.4. Eci1 Knockout Partially Counteracts the Benefits of Exercise

**Figure 4:**
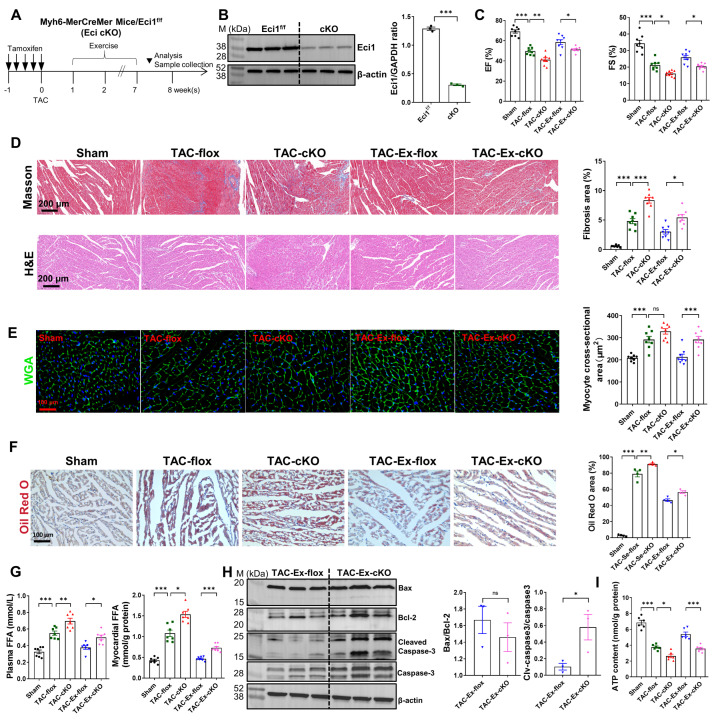
The role of *Eci1* in ameliorating heart failure by exercise intervention. (**A**) Schematic diagram of exercise intervention. Arrows indicate timing of tamoxifen administration. (**B**) Verification of *Eci1* knockdown efficiency in myocardial tissues (*n* = 3 per group). (**C**) Cardiac function was evaluated by calculating EF and FS (*n* = 8 per group). EF, ejection fraction; FS, fractional shortening. (**D**) Representative images of Masson’s trichrome staining and H&E staining of heart tissue. Quantitative results of Masson’s trichrome staining (*n* = 8 per group). Scale bar, 200 µm. (**E**) Wheat germ agglutinin (WGA, green) staining showed cardiomyocyte membrane staining and quantification results for the cardiomyocyte cross sectional area (*n* = 8 per group). Scale bar, 100 µm. (**F**) Oil Red O staining and lipid content quantification (*n* = 4 per group). Scale bar, 100 µm. (**G**) FFA levels in plasma and myocardial tissues (*n* = 7 per group). FFA, free fatty acid. (**H**) Apoptosis related proteins were detected by Western blot (*n* = 3 per group). (**I**) Relative ATP content of myocardial tissues (normalized using protein concentration, *n* = 6 per group). * *p* < 0.05, ** *p* < 0.01, *** *p* < 0.001, ns indicates *p* > 0.05.

To explore the role of Eci1 in the progression of TAC-induced heart failure in vivo, we employed a cardiac-specific *Eci1* knockout mouse model (*Eci1*-cKO) (Supplementary Figure S1A). All mice received tamoxifen one week prior to the surgical procedure (Figure 4A). Western blot analysis was utilized to assess the efficiency of Eci1 knockout (Figure 4B). Mice assigned to the exercise group participated in a six-week exercise regimen (Figure 4A). As depicted in Supplementary Figure S2A, *Eci1* knockout resulted in an increase in HW/BW and HW/TL ratios compared with each control group. Notably, the TAC-Ex-cKO mice exhibited a significant increase in HW/BW and HW/TL ratios compared to the TAC-Ex-flox group. Additionally, myocardial hypertrophy markers, including ANP, BNP, and MYH7, were significantly elevated in TAC-cKO mice relative to TAC-flox mice. Furthermore, Eci1 knockout enhanced the expression of ANP and BNP in the exercise group of mice (Supplementary Figure S2B). Echocardiographic assessments similarly indicated that *Eci1* knockout exacerbated cardiac dysfunction in TAC mice and partially negated the beneficial effects of exercise (Figure 4C). However, there was no significant differences were observed in EF and FS values prior to modeling (Supplementary Figure S1B). Histological staining analysis demonstrated that *Eci1* knockout facilitated myocardial fibrosis and partially mitigated the benefits of exercise (Figure 4D). Correspondingly, WGA staining confirmed that *Eci1* knockout led to an increased cross-sectional area of cardiomyocytes (Figure 4E). Furthermore, Oil Red O staining and FFA assays revealed that *Eci1* knockout led to elevated lipid deposition and FFA levels in myocardial tissues (Figure 4F,G). These findings indicated that *Eci1* deficiency resulted in abnormal lipid metabolism and partially abolished exercise effects. Recent research has implicated apoptosis in the progression of myocardial hypertrophy [42]. Therefore, we examined whether *Eci1* knockout in exercise group mice would aggravate the apoptosis. Our results showed no significant difference in the Bax/Bcl2 ratio; however, there was a notable increase in the Cleaved-Caspase3/Caspase3 ratio (Figure 4H). To evaluate the impact of *Eci1* knockout on mitochondrial function, we quantified ATP content. The results revealed a significant reduction in ATP content within the myocardial tissues of both TAC and TAC-Ex mice post-*Eci1* knockout (Figure 4I). In summary, the absence of *Eci1* appears to facilitate the progression of pathological myocardial hypertrophy, cellular apoptosis, and myocardial fibrosis induced by TAC, while partially negating the beneficial effects of exercise.

### 3.5. Eci1 Displays a Protective Role Against Cardiomyocyte Hypertrophy

**Figure 5:**
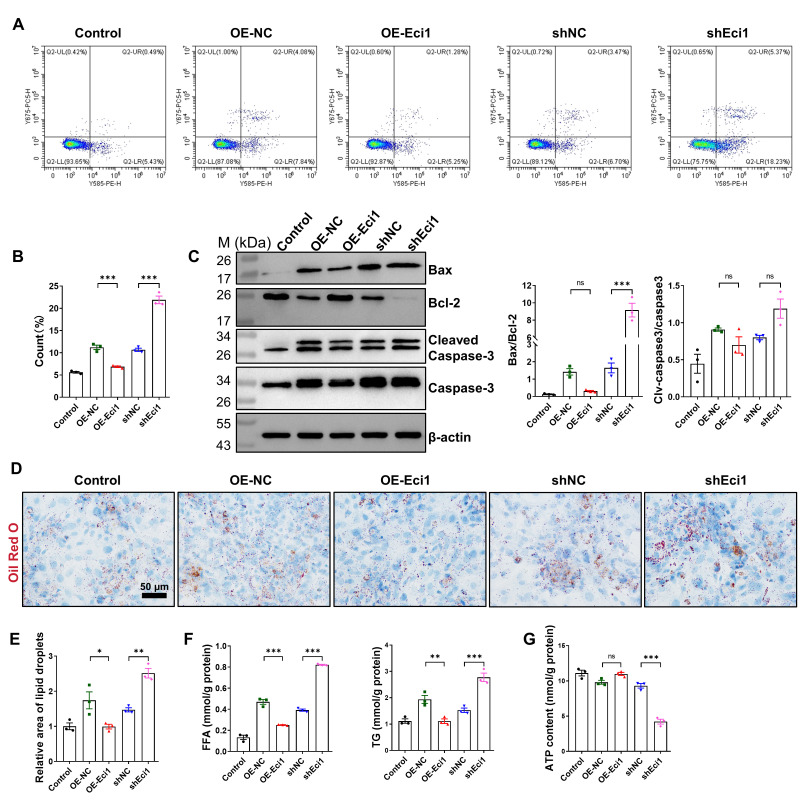
The role of Eci1 in cardiomyocyte hypertrophy induced by Ang II. (**A**) Detection of cell apoptosis by flow cytometry analysis. (**B**) The statistic results of cell apoptosis by flow cytometry (*n* = 3 per group). (**C**) The apoptosis related proteins were detected (*n* = 3 per group). (**D**) Representative image of Oil Red O stain. (**E**) Evaluation of lipid accumulation by Oil Red O staining (*n* = 3 per group). Scale bar, 50 µm. (**F**) FFA levels and TG content in each group (*n* = 3 per group). FFA, free fatty acid; TG, triglyceride. (**G**) Relative ATP content of cardiomyocytes (normalized using protein concentration, *n* = 3 per group). Statistical significance is denoted by: * *p* < 0.05, ** *p* < 0.01, *** *p* < 0.001, ns indicates *p* > 0.05. OE-NC, overexpression-control. OE-Eci1, overexpression-Eci1. shNC, shRNA-control. shEci1, shRNA-Eci1.

To establish a model of cell hypertrophy, primary cardiomyocytes were isolated from neonatal mice (Supplementary Figure S3A,C). These cells were treated with various concentrations of Ang II (0, 2, 6, 10, 14 μM) for 48 h to determine the optimal conditions for hypertrophy induction. The CCK8 assay identified 10 μM as the most effective concentration (Supplementary Figure S3B). Lentiviral transduction was employed to achieve either overexpression or knockdown of Eci1. The efficiency of the lentiviral transfection was validated via qRT-PCR analysis (Supplementary Figures S4A,B). It was observed that the inhibition of Eci1 significantly elevated the mRNA expression levels of *ANP*, *BNP*, and *MYH7*, which are recognized markers of myocardial hypertrophy. Conversely, Eci1 overexpression significantly decreased the mRNA expression levels of these markers (Supplementary Figure S5A). Previous research has highlighted the critical role of cardiomyocyte proliferation in pathological myocardial hypertrophy [43]. This prompted an investigation into the potential impact of Eci1 on cardiomyocyte proliferation. Utilizing lentiviral vectors, we modulated Eci1 expression in cardiomyocytes and conducted a CCK8 assay. The results demonstrated a notable increase in cardiomyocyte proliferation subsequent to Eci1 overexpression, whereas Eci1 knockdown significantly reduced proliferation (Supplementary Figure S5B). Immunofluorescence double-staining for Ki67 and cTnT indicated that Eci1 overexpression led to a higher percentage of Ki67+ cTnT+ cells, signifying enhanced proliferation of primary cardiomyocytes. In contrast, Eci1 knockdown was associated with a suppression of primary cardiomyocyte proliferation (Supplementary Figure S5C). Moreover, flow cytometry analysis demonstrated that Eci1 overexpression inhibited apoptosis in primary cardiomyocytes, while Eci1 suppression increased apoptosis in these cells (Figure 5A,B). Consistent with the flow cytometry findings, an elevated Bax/Bcl2 ratio was observed at the protein level in primary cardiomyocytes following Eci1 suppression. However, there were no significant differences in the expression levels of Cleaved-Caspase3/Caspase3 (Figure 5C). Additionally, Eci1 knockdown led to a significant increase the production of FFA, TG, as well as the accumulation of lipid droplets (Figure 5D–F). To assess cellular ATP levels, cardiomyocytes were harvested and their ATP content was quantified. The results demonstrated that Eci1 knockdown impaired mitochondrial function, as evidenced by a reduction in ATP production (Figure 5G). Our findings suggest that Eci1 is essential for promoting proliferation, inhibiting apoptosis, and mitigating lipid deposition in primary cardiomyocytes under Ang II stimulation.

### 3.6. FTO Regulates Eci1 Expression in an m^6^A Dependent Manner

**Figure 6:**
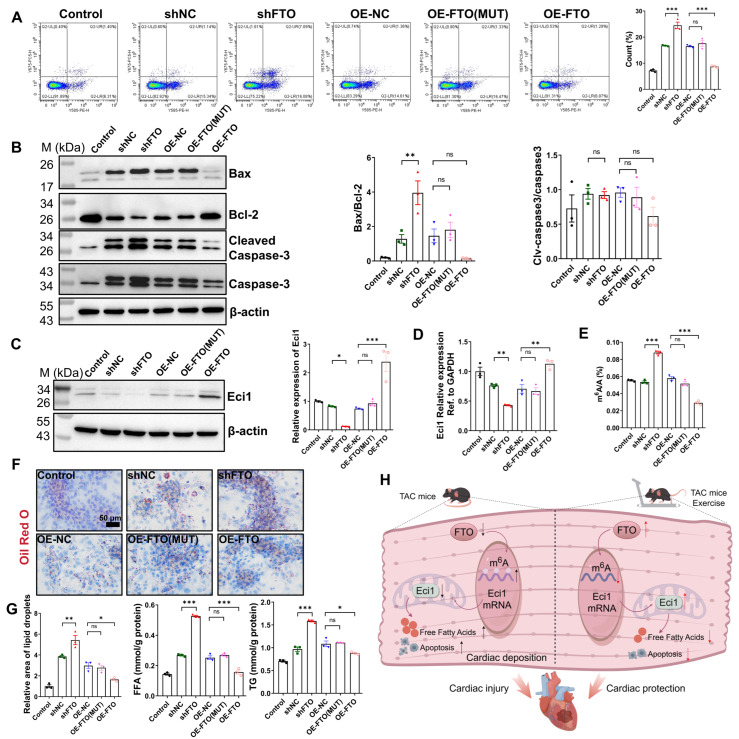
FTO promoted the expression of Eci1 dependent on its m^6^A demethylase activity. (**A**) Flow cytometry analysis was used to detect cell apoptosis (*n* = 3 per group). (**B**) Apoptosis related proteins were detected by Western blot (*n* = 3 per group). (**C**) Eci1 protein expression was demonstrated by Western blot (*n* = 3 per group). (**D**) The expression of *Eci1* was measured by qRT-PCR. (**E**) Total m^6^A content was detected. (**F**) Oil Red O staining and lipid content quantification (*n* = 3 per group). Scale bar, 50 µm. (**G**) FFA levels and TG content in each group (*n* = 3 per group). FFA, free fatty acid; TG, triglyceride. (**H**) Diagram of the proposed molecular mechanism of the cardioprotective effect of exercise intervention. The downward arrow denotes a decrease, whereas the upward arrow denotes an increase in content, production, or expression. The diagram was drawn by using Figdraw (www.figdraw.com). Statistical significance is denoted by: * *p* < 0.05, ** *p* < 0.01, *** *p* < 0.001, ns indicates *p* > 0.05. shNC, shRNA-control. shEci1, shRNA-FTO. OE-NC, overexpression-control. OE-FTO, overexpression-FTO. OE-FTO (MUT), overexpression-FTO (mutated FTO, R96Q).

In this section, we utilized the same cardiomyocyte hypertrophy model. Lentiviral transduction was employed to facilitate either the overexpression or knockdown of FTO, with qRT-PCR analysis confirming the successful transfection efficiency of the lentiviral infection (Supplementary Figure S6A,B). Given that FTO functions as an m^6^A demethylase, our objective was to determine whether its demethylase activity on m^6^A is essential for the regulation of Eci1 expression. To investigate this, we mutated the FTO residue R96 to Q96, generating a mutated FTO (R96Q, FTO-MUT) with impaired demethylase activity. As illustrated in Supplementary Figure S7A, FTO knockdown led to increased mRNA expression levels of *ANP*, *BNP*, and *MYH7*, whereas FTO overexpression resulted in reduced expression levels of these markers. Notably, the overexpression of FTO-MUT partially attenuated the anti-hypertrophic effects of FTO overexpression (Supplementary Figure S7A). Cell proliferation assays, conducted using CCK-8 and immunofluorescence double-staining, indicated that FTO knockdown suppressed cell proliferation activity. Conversely, FTO overexpression enhanced cell proliferation activity, although this proliferative capacity was partially inhibited by FTO-MUT overexpression (Supplementary Figure S7B,C). Furthermore, flow cytometry and Western blot analysis of apoptosis-associated proteins were employed to evaluate the apoptotic condition. The findings indicate that FTO knockdown enhanced Ang II-induced apoptosis, whereas FTO overexpression attenuated apoptosis. Notably, the anti-apoptotic effect was partially inhibited by the overexpression of FTO-MUT (Figure 6A,B). While significant differences were observed in the Bax/Bcl2 ratio, no significant differences were found in the Cleaved-Caspase3/Caspase3 ratio among the groups compared (Figure 6B). In addition, Western blot analysis confirmed the downregulation of Eci1 following FTO knockdown, whereas Eci1 expression was upregulated in cells with FTO overexpression. Notably, FTO-MUT cells restored Eci1 expression to levels comparable to the control group (Figure 6C). As depicted in Figure 6D, the qRT-PCR results for *Eci1* expression were consistent with the Western blot findings. Furthermore, we observed a significant increase in mRNA m^6^A levels in cardiomyocytes upon FTO knockdown, whereas FTO overexpression led to a marked decrease in m^6^A levels. FTO-MUT cells restored the m^6^A levels to those of the control group (Figure 6E). Additionally, the levels of FFA and TG, as well as lipid droplet accumulation in cardiomyocytes, were significantly increased in cells with FTO knockdown. In contrast, these levels were markedly decreased with FTO overexpression. Moreover, FTO-MUT overexpression resulted in a reduction FFA and TG levels and decreased lipid droplet accumulation (Figure 6F,G). These observations indicate that FTO regulates Eci1 expression via m^6^A methylation, thereby influencing cellular proliferation, apoptosis, and lipid accumulation. Taken together, our results demonstrate that exercise upregulates FTO expression and diminishes m^6^A modification of *Eci1*, promoting its expression. This process ultimately leads to reduced lipid deposition and apoptosis, contributing to a cardioprotective effect, as illustrated in Figure 6H.

## 4. Discussion

Exercise interventions have been demonstrated to exert significant effects on various diseases, including cardiovascular disease. Exercise-induced cardiac adaptations result in alterations in metabolites and metabolism [44,45]. Previous research has indicated that endurance exercise training leads to the downregulation of cardiac METTL14 expression, consequently reducing mRNA m^6^A levels [46]. Furthermore, aerobic exercise training has been shown to mitigate ischemia–reperfusion injury in mice by decreasing the methylation level of METTL3-related m^6^A RNA in cardiomyocytes [47]. Recent studies have highlighted the critical role of the m^6^A methyltransferase METTL3 in mediating the cardiovascular benefits of exercise and its potential in preventing diabetic cardiomyopathy [48]. These findings provide compelling evidence for the relationship between exercise and m^6^A modifications. In line with the objective of this study, we focus on pathological cardiac hypertrophy and heart failure. It has been reported that m^6^A levels are elevated in heart failure, hypertrophic hearts, and isolated cardiomyocytes under stress conditions [6,7,49]. In this study, we demonstrated that exercise intervention provides protection against heart failure, while concurrently enhancing cardiac FTO expression and reducing mRNA m^6^A methylation levels in myocardial tissues. The beneficial effects of exercise were partially diminished upon FTO inhibition. Subsequently, the target gene Eci1 was identified through MeRIP-seq and RNA-seq analyses. Furthermore, the role of Eci1 was investigated in vivo utilizing a transgenic mouse model and a primary cardiomyocyte model. Finally, we validated the function of Eci1 in FTO-dependent demethylase regulation at the cellular level. Mechanistically, FTO modulated the fatty acid metabolism process mediated by Eci1 in an m^6^A-dependent manner, playing a crucial regulatory role in exercise-induced cardioprotection in heart failure.

The m^6^A modification is catalyzed by the METTL3-METTL14-WTAP methyltransferase complex, commonly referred to as the “writer” complex [50], and can be reversed by m^6^A demethylases such as FTO [13] and ALKBH5, known as “erasers” [51]. Previous research has demonstrated that FTO mitigates cardiac fibrosis following myocardial infarction through m^6^A -mediated epigenetic modifications [52]. In the present study, we provided robust in vitro and in vivo evidence underscoring the significant role of FTO in myocardial hypertrophy in mice subjected to TAC surgery and in cardiomyocyte hypertrophy induced by Ang II stimulation. Our observations indicated that exercise effectively ameliorated cardiac dysfunction, inhibited myocardial hypertrophy, and reduced myocardial fibrosis in response to the mechanical overload of TAC. Furthermore, the study revealed that exercise intervention led to a reduction in the m^6^A modification levels of total RNA in hypertrophic myocardial tissues, potentially associated with alterations in the expression of the demethylase FTO. Although it has been previously reported that FTO alleviates cardiac dysfunction by modulating glucose uptake and glycolysis in mice with pressure overload-induced heart failure [21], the role of FTO in mitigating TAC-induced heart failure through exercise intervention remains unexplored. Western blot and qRT-PCR analyses revealed a significant increase in FTO expression in the TAC-Ex group. Recent studies have highlighted the beneficial effects of upregulated demethylase FTO expression in the hearts of mice with heart failure, particularly in enhancing cardiac systolic function [7]. Consistent with these findings, FTO knockdown in murine hearts resulted in impaired cardiac function, exacerbated hypertrophy, increased fibrosis, and partially negated the benefits of exercise. This underscores the critical role of m^6^A RNA methylation, regulated by FTO, in maintaining normal cardiac function. Furthermore, our in vitro experiments corroborated that FTO overexpression promoted cell proliferation and reduced cardiomyocyte apoptosis. Collectively, our findings elucidate the protective role of FTO in cardiac function and remodeling in mice with heart failure, with exercise serving as an intervention to alleviate heart failure progression.

Furthermore, we focused on the profiles of altered m^6^A-modified transcripts and gene expression following TAC-induced hypertrophy and subsequent exercise intervention. Our findings provide evidence that exercise training influences epigenetic regulation through an RNA-related mechanism, thereby enhancing our understanding of the regulation of cardiac homeostasis and pathogenesis via m^6^A modification. Analysis of the distribution profiles of m^6^A peaks within mRNAs in myocardial tissues revealed that m^6^A sites were predominantly located in coding sequences and stop codon regions. This observation was corroborated by differential m^6^A modifications identified in the MeRIP-seq data, suggesting that the impact of m^6^A modification on myocardial hypertrophy may be associated with its role in modulating processes such as translation [53]. KEGG pathway analysis revealed that the differentially methylated genes were associated with fatty acid metabolism. Through an integrated analysis of MeRIP-seq and RNA-seq data, we identified *Eci1* as a potential target gene. This gene demonstrated hyper-methylation and was downregulated in the TAC mouse model. However, subsequent to exercise intervention in TAC mice, *Eci1* expression increased with hypo-methylation. These observations were substantiated by MeRIP-qPCR, qRT-PCR, and Western blot analyses. Eci1, as a key enzyme in the fatty acid metabolism pathway, plays a critical role in the synthesis and metabolism of unsaturated fatty acids, which are essential for intracellular energy regulation and lipid metabolism [23]. Additionally, we observed elevated levels of FFA and TG in the myocardial tissue and plasma of TAC mice, indicating that persistent pressure overload disrupts lipid metabolism. Notably, this metabolic abnormality was reversed by exercise training.

The role of Eci1 in fatty acid metabolism and its potential impact on the exercise-induced amelioration of cardiac hypertrophy remains sufficiently elucidated. In the present study, we observed an increase in lipid accumulation and FFA levels in the myocardial tissues of *Eci1* cKO mice following TAC surgery. Notably, these alterations were significantly attenuated by exercise. Recent research has shown that in the hearts and plasma of mice with diabetic cardiomyopathy, key genes, including *Eci1* and *Eci2*, etc., are abnormally regulated, leading to branched-chain amino acids and fatty acid disorders [54]. *Eci2* is localized in both mitochondria and peroxisomes, whereas *Eci1* is confined to mitochondria [55]. Another study identified Eci1 as a potential biomarker for post-infarction complications in diabetic patients [56]. Furthermore, *Eci1* knockdown has been reported to inhibit cardiomyocyte growth under chronic hypoxic conditions [57]. Importantly, our findings are the first to demonstrate that *Eci1* knockout significantly impairs cardiac function and mitochondrial ATP production, while exacerbating fibrosis and promoting apoptosis in TAC mice. Besides, the knockout of *Eci1* partially counteracted the benefits of exercise, suggesting that *Eci1* may serve as a potential therapeutic target. It is crucial to highlight, however, that the EF and FS values in both control and *Eci1* knockout mice did not display significant differences prior to TAC surgery. This observation can be attributed to the mitochondrial dysfunction induced by pressure overload from TAC surgery [58], which *Eci1* knockout mice are unable to effectively manage due to the absence of the protective *Eci1* gene, ultimately leading to compromised cardiac function. Our findings indicate that Eci1 plays a novel role in fatty acid metabolism and the maintenance of cardiac homeostasis. Under normoxic conditions, fatty acids constitute the primary energy source, and adaptive mechanisms are essential for the survival and growth of cardiomyocytes. This study found that Ang II-induced hypertrophic cardiomyocytes showed increased cell proliferation, enhanced mitochondrial ATP production, and reduced apoptosis with Eci1 overexpression, whereas Eci1 knockdown resulted in the opposite effects. The current study corroborates previous research suggesting that Eci1 overexpression promotes the proliferation of prostate cancer cells, while Eci1 deficiency impedes their growth [59]. Additionally, Eci1 knockdown led to an increase in FFA and TG, as well as the accumulation of lipid droplets in Ang II-induced hypertrophic cardiomyocytes, whereas Eci1 overexpression alleviated these effects. These findings highlight the pivotal role of Eci1-mediated fatty acid metabolism in cardiomyocytes. Importantly, the study revealed that Eci1 expression levels were upregulated by FTO overexpression, as evidenced by Western blot analysis, while FTO knockdown suppressed Eci1 expression. Nonetheless, the specific mechanisms through which FTO regulates Eci1 expression warrant further detailed investigation.

Mechanistically, our data indicate that FTO enhances Eci1 expression in cardiomyocytes through an m^6^A-dependent mechanism. The critical role of m^6^A modification in tissue and cellular injury has been acknowledged in several studies. For instance, recent research has underscored the importance of the m^6^A-modified planar cell polarity pathway in regulating postnatal lens fiber organization, suggesting its potential as a therapeutic target for highly myopic cataracts [60]. Moreover, the restoration of m^6^A homeostasis via small molecule intervention or gene therapy has been demonstrated to significantly protect motor neurons from degeneration and alleviate motor impairments in amyotrophic lateral sclerosis models, including induced pluripotent stem cell-derived motor neurons and murine models [61]. Additionally, ELF1-mediated transactivation of METTL3/YTHDF2 has been found to promote nucleus pulposus cell senescence through m^6^A-dependent destabilization of *E2F3* mRNA in intervertebral disc degeneration [62]. Our findings align with these studies and underscore the essential role of m^6^A modification in the regulation of tissue damage. Our functional rescue assays demonstrated that FTO promotes cardiomyocyte growth and inhibits apoptosis upon exposure to Ang II, which is associated with elevated levels of Eci1 and reduced m^6^A methylation. Conversely, inhibition of FTO demethylase activity by FTO^R96Q^ appeared to counteract these effects, promoting cardiomyocyte apoptosis and inhibiting growth, alongside reductions in Eci1 and increased m^6^A levels. Additionally, lipid accumulation in hypertrophic cardiomyocytes, as well as FFA and TG levels, were influenced by alterations in Eci1 expression. Specifically, FTO overexpression led to increased Eci1 expression and subsequently reduced lipid content. Our findings indicate that FTO modulates Eci1 expression through an m^6^A-dependent mechanism in Ang II-induced hypertrophic cardiomyocytes.

Collectively, our findings support Eci1 as a pivotal regulator of lipid metabolism in the heart. The observed accumulation of FFA and TG following *Eci1* knockout under conditions of prolonged pressure overload highlights its essential function in maintaining fatty acid homeostasis. Therapeutically, restoring Eci1 activity may offer a novel approach for reducing lipid accumulation and improving cardiac dysfunction. Finally, there are limitations in our research that are worth addressing and improving. First, we did not perform serial echocardiograms during the exercise training period, limiting our ability to capture dynamic changes in cardiac contractility indicators. Second, our research target focus on mitochondrial coenzyme Eci1, but we did not perform experiments on isolated mitochondria, which may have provided additional mechanistic insights. Future research should incorporate longitudinal monitoring and isolated mitochondrial assays to address these gaps.

## 5. Conclusions

In the present study, we identified elevated levels of FTO and Eci1, accompanied by decreased levels of m^6^A, during the pathological progression of heart failure. Our findings indicate that FTO-mediated m^6^A modification plays a positive regulatory role in the expression of Eci1 within the context of myocardial hypertrophy. Notably, the inhibition of FTO expression or the knockout of *Eci1* during exercise in TAC mice partially counteract the beneficial effects of exercise, suggesting that FTO-mediated regulatory mechanisms are essential for the amelioration of heart failure through exercise. In conclusion, our study demonstrates that FTO modulates the m^6^A modification of *Eci1* mRNA in cardiomyocytes, thereby affecting Eci1 expression and influencing fatty acid metabolism. This finding elucidates the molecular mechanism by which RNA m^6^A modification alleviates heart failure through exercise. Targeting the FTO-mediated m^6^A modification of Eci1 presents a promising therapeutic strategy for addressing heart failure in mammals.

## Figures and Tables

**Table 1:** The name and sequence of the lentiviral vector.

Plasmid	Group	Expressed Sequence
LV-EF1a-EGFP-CMV-Puro-WPRE	OE-NC	/
LV-EF1a-Eci1-EGFP-CMV-Puro-WPRE	OE-Eci1	NM_010023.4
LV-EF1a-FTO(wt)-EGFP-CMV-Puro-WPRE	OE-FTO	NM_011936.2
LV-EF1a-FTO(mut)-EGFP-CMV-Puro-WPRE	OE-FTO(MUT)	NM_011936.2 (R96Q)
LV-U6-shRNA(NC)-CMV-EGFP-T2A-Puro-WPRE	shNC	CCTAAGGTTAAGTCGCCCTCG
LV-U6-shRNA(Eci1)-CMV-EGFP-T2A-Puro-WPRE	shEci1	GCTGACAACCCCAAATATACT
LV-U6-shRNA(FTO)-CMV-EGFP-T2A-Puro-WPRE	shFTO	GCACCTTGGATTATATCTTAG

## Data Availability

MeRIP-seq and RNA-seq data have been deposited at NCBI BioProject with the accession number PRJNA1211335.

## Supplementary Materials

The following supporting information can be downloaded at: https://www.mdpi.com/article/10.3390/biom16010098/s1, Figure S1: Diagram of *Eci1*-knockout mice construction strategy; Figure S2: The effect of *Eci1* knockout on heart weight and myocardial hypertrophy markers; Figure S3: Screening of the optimal concentration of Ang II in inducing cardiomyocyte hypertrophy; Figure S4: Eci1 overexpression and knockdown in cardiomyocyte was achieved by lentivirus transduction; Figure S5: The effect of Eci1 inhibition or overexpression on myocardial hypertrophy markers and cardiomyocyte proliferation; Figure S6: FTO overexpression and knockdown in cardiomyocyte was achieved by lentivirus transduction; Figure S7: The effect of FTO inhibition or overexpression on myocardial hypertrophy markers and cardiomyocyte proliferation. File S1. Original WB.

## Abbreviations

The following abbreviations are used in this manuscript:

AAV	Adeno-associated virus
Ang II	Angiotensin II
BW	Body weight
Eci1	Enoyl-CoA delta isomerase 1
FFA	Free fatty acid
FTO	Fat mass and obesity-associated protein
HW	Heart weight
m^6^A	N6-methyladenosine
qRT-PCR	Quantitative real-time PCR
RIP	RNA immunoprecipitation
TAC	Transverse aortic constriction
TG	Triglyceride
TL	Tibia length
