# Supplementary material for: FTO-Eci1 Axis Mediates Exercise-Induced Cardioprotection in Pressure Overload Mice
Source: Biomolecules. 2026 Jan 7;16(1):98. doi: 10.3390/biom16010098 (PMC12839096; doi:10.3390/biom16010098)
Supplement: Supplementary file 1 [file biomolecules-16-00098-s001.zip › biomolecules-4027548-supplementary.pdf]

Supplementary information

FTO-Eci1 axis mediates exercise-induced cardioprotection in pressure overload mice

Jinyun Wang, Zaoshang Chang, Shuo Lin, Guangyuan Sha, Wenyan Zeng, Qirong Huang, Qibin Deng, Shen Wang, Min Hu, Jingbo Xia

Supplementary Figure S1. Diagram of Eci1-knockout mice construction strategy.
Supplementary Figure S2. The effect of Eci1 knockout on heart weight and myocardial hypertrophy markers.
Supplementary Figure S3. Screening of the optimal concentration of Ang II in inducing cardiomyocyte hypertrophy.
Supplementary Figure S4. Eci1 overexpression and knockdown in cardiomyocyte was achieved by lentivirus transduction.
Supplementary Figure S5. The effect of Eci1 inhibition or overexpression on myocardial hypertrophy markers and cardiomyocyte proliferation.
Supplementary Figure S6. FTO overexpression and knockdown in cardiomyocyte was achieved by lentivirus transduction.
Supplementary Figure S7. The effect of FTO inhibition or overexpression on myocardial hypertrophy markers and cardiomyocyte proliferation.

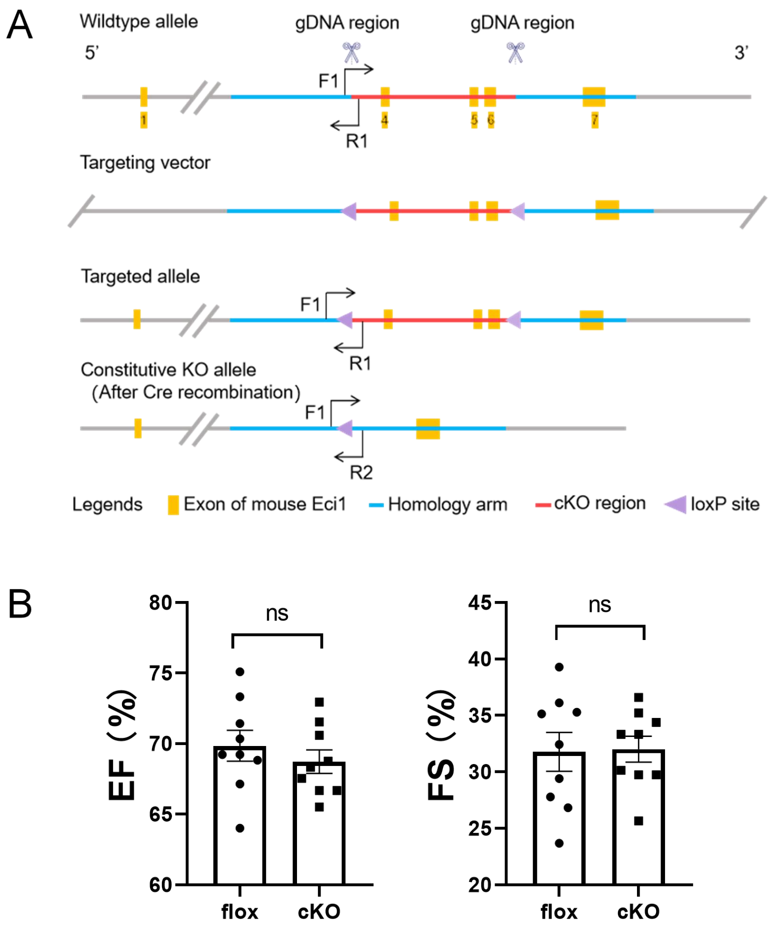

Supplementary Figure S1. Diagram of Eci1-knockout mice construction strategy. (A) Schematic of mouse Eci1 gene, targeting construct, and null allele. (B) The evaluation of cardiac function was detected by echocardiography. The EF and FS were then both calculated (n = 9 per group). 14 week old male mice were subjected to echocardiography. This time-point was corresponding to TAC-induced heart failure. EF, ejection fraction; FS, fractional shortening. Data are presented as Mean ± SEM.

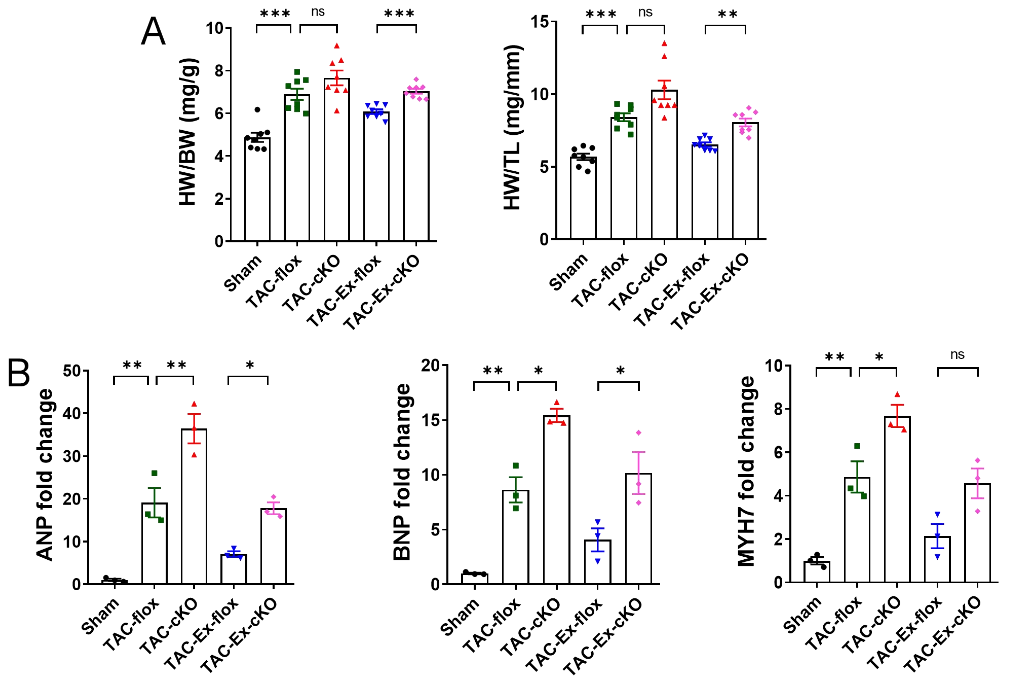

Supplementary Figure S2. The effect of Eci1 knockout on heart weight and myocardial hypertrophy markers. (A) HW/BW and HW/TL for the indicated groups (n = 8 per group). BW, body weight; HW, heart weight; TL, tibia length. (B) The cardiac hypertrophy biomarkers ANP, BNP, and MYH7 levels were measured using qRT-PCR (n = 3 per group). * $P < 0.05$, ** $P < 0.01$, *** $P < 0.001$.

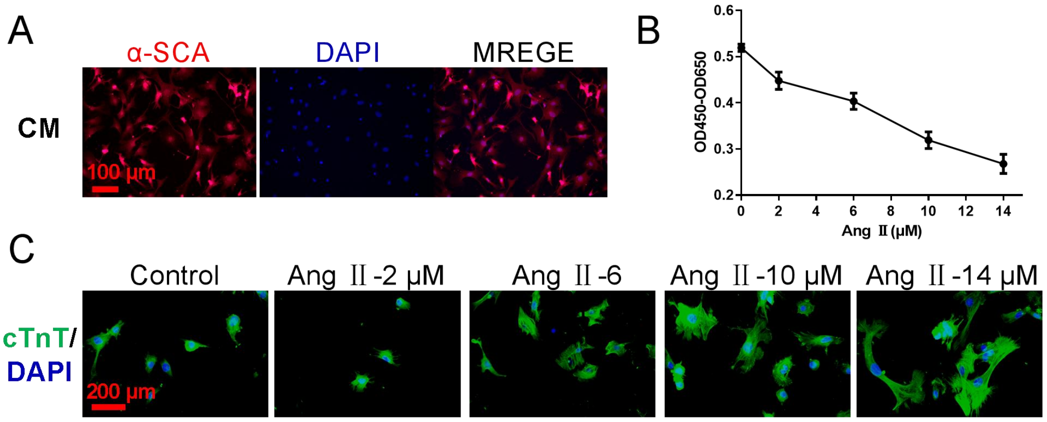

Supplementary Figure S3. Screening of the optimal concentration of Ang II in inducing cardiomyocyte hypertrophy. (A) Cardiomyocyte identification Immunofluorescent staining of α-SCA (red) was performed 48 hr after cell isolation to identify cardiomyocytes. (B) the cell proliferation inhibition ratio was assessed by CCK8 (n = 3 per group). (C) Cardiomyocyte hypertrophy was evaluated by anti-cTnT (green) immunofluorescence staining. Data are presented as Mean ± SEM.

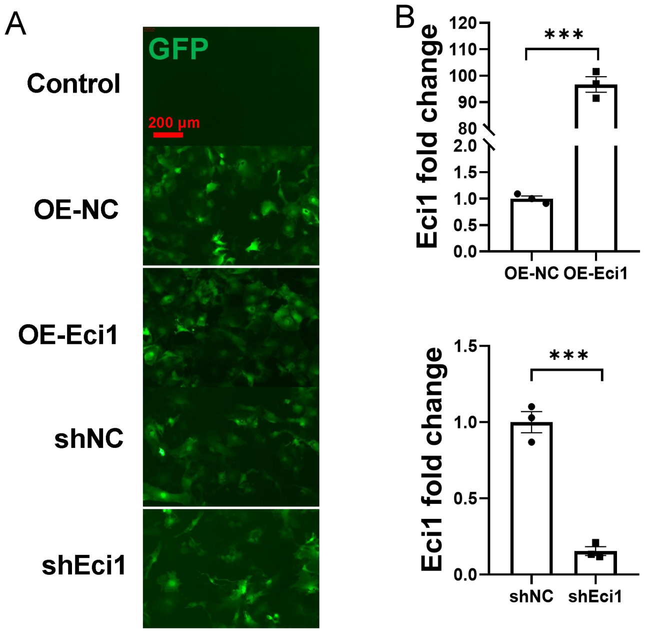

Supplementary Figure S4. Eci1 overexpression and knockdown in cardiomyocyte was achieved by lentivirus transduction. (A) The efficiency of Eci1 overexpression and knockdown by lentiviral transfection was determined through EGFP positive (green) cells observed via fluorescence microscopy. (B) The lentiviral transfection efficiency was detected by qRT-PCR (n = 3 per group). Data are presented as Mean ± SEM, $***P < 0.001$. OE-NC, overexpression-control. OE-Eci1, overexpression-Eci1. shNC, shRNA-control. shEci1, shRNA-Eci1.

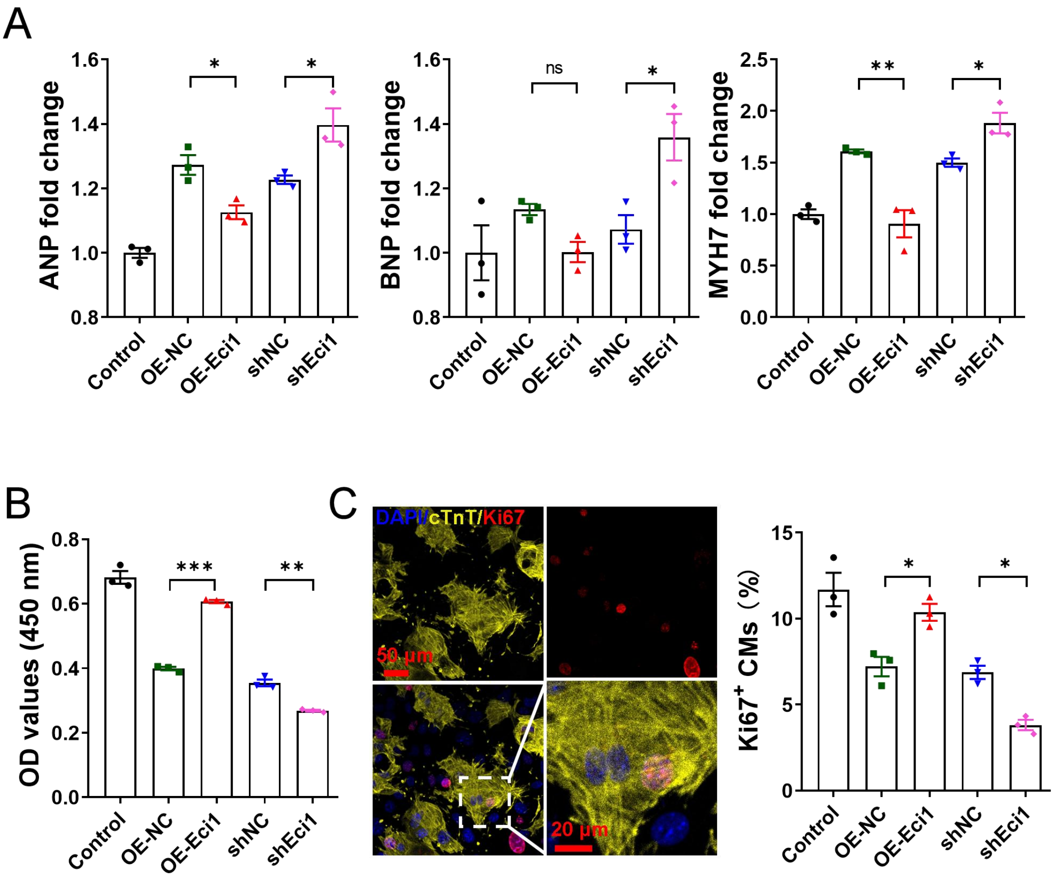

Supplementary Figure S5. The effect of Eci1 inhibition or overexpression on myocardial hypertrophy markers and cardiomyocyte proliferation. (A Validation of mRNA expression levels of cardiac hypertrophy marker ANP, BNP, and MYH7 after incubated with Ang II (n = 3 per group. (B Cell viability was evaluated by testing the OD 45nm (n = 3 per group. (C Cell proliferation marker Ki67 was evaluated. Ki67+ cTnT+ cells (proliferating cardiomyocytes were counted (n = 3 per group. Ki67 (red. Cardiac troponin T (cTnT (yellow, DAPI (blue. * $P < .5$, ** $P < .1$, *** $P < .1$.

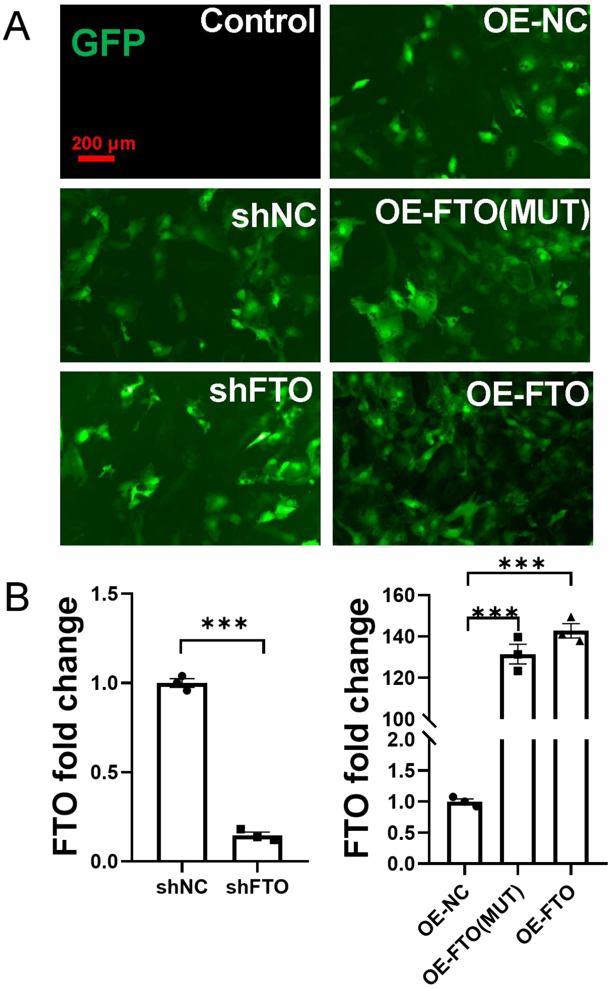

Supplementary Figure S6. FTO overexpression and knockdown in cardiomyocyte was achieved by lentivirus transduction. (A) The efficiency of FTO overexpression and knockdown by lentiviral transfection was determined through EGFP positive (green) cells observed via fluorescence microscopy. (B) The lentiviral transfection efficiency was detected by qRT-PCR (n = 3 per group). Data are presented as Mean ± SEM, ****P* < 0.001. shNC, shRNA-control. shEci1, shRNA-FTO. OE-NC, overexpression-control. OE-FTO, overexpression-FTO. OE-FTO (MUT), overexpression-FTO (mutated FTO, R96Q).

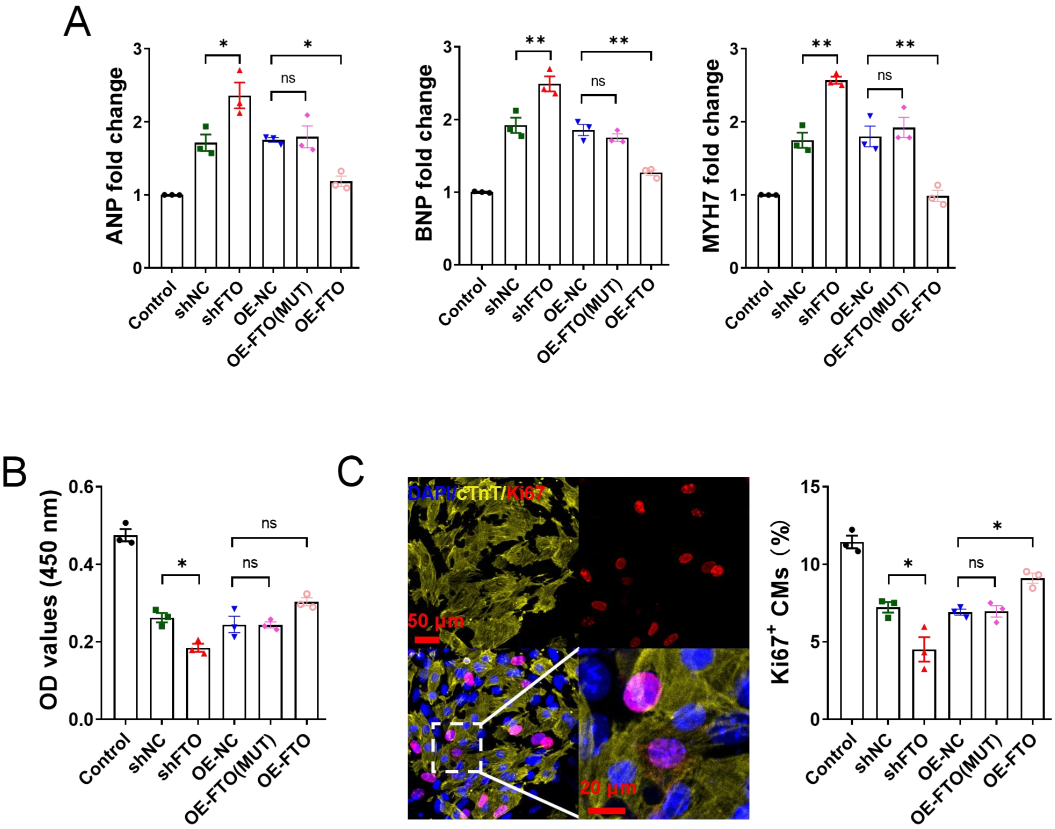

Supplementary Figure S7. The effect of FTO inhibition or overexpression on myocardial hypertrophy markers and cardiomyocyte proliferation. (A) The cardiac hypertrophy biomarkers ANP, BNP, and MYH7 levels were measured using qRT-PCR (n = 3 per group). (B) Cell proliferation was measured (n = 3 per group). (C) Cell proliferation marker Ki67 was evaluated. Ki67+ cTnT+ cells (proliferating cardiomyocytes) were counted (n = 3 per group). Ki67 (red). Cardiac troponin T (cTnT) (yellow), DAPI (blue). * $P < 0.05$, ** $P < 0.01$.
