# Supplementary figures and images for: FTO-Eci1 Axis Mediates Exercise-Induced Cardioprotection in Pressure Overload Mice
Source: Biomolecules. 2026 Jan 7;16(1):98. doi: 10.3390/biom16010098 (PMC12839096; doi:10.3390/biom16010098)

Figure 1H

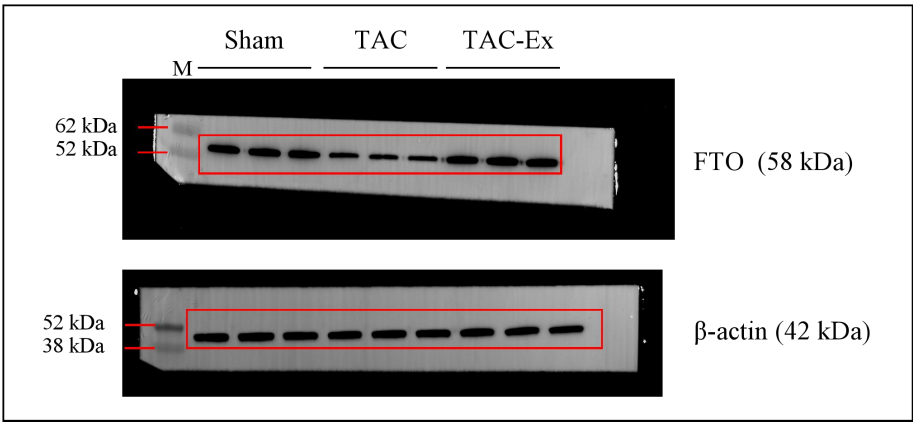

Figure 3J

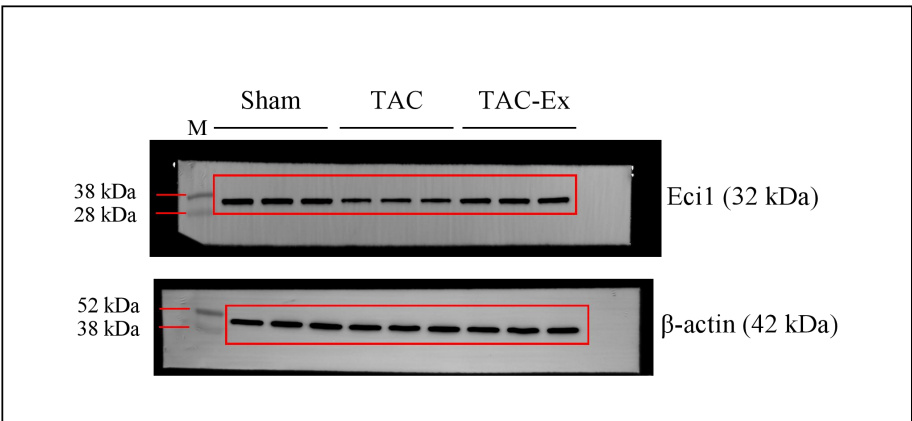

Figure 4B

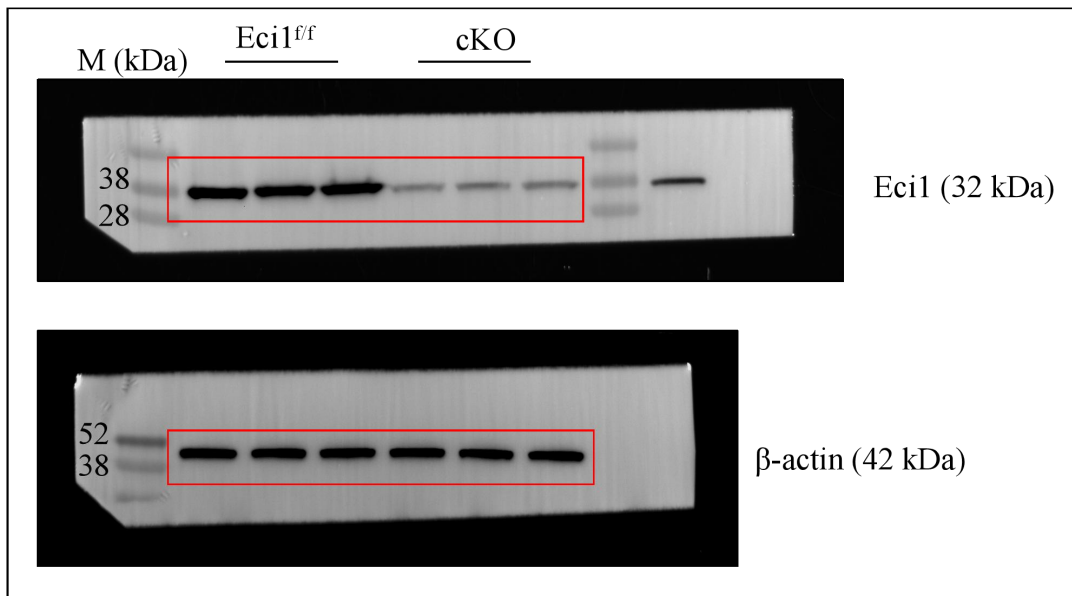

Figure 4J

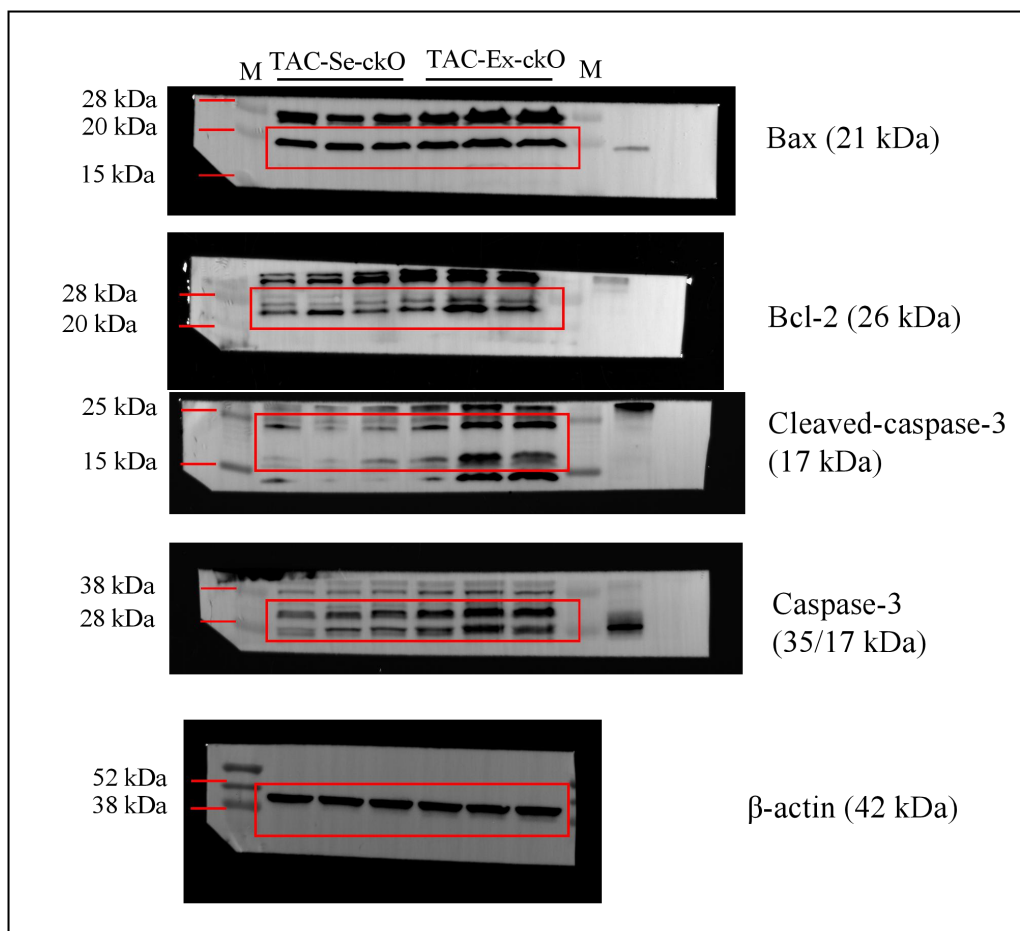

Figure 5F

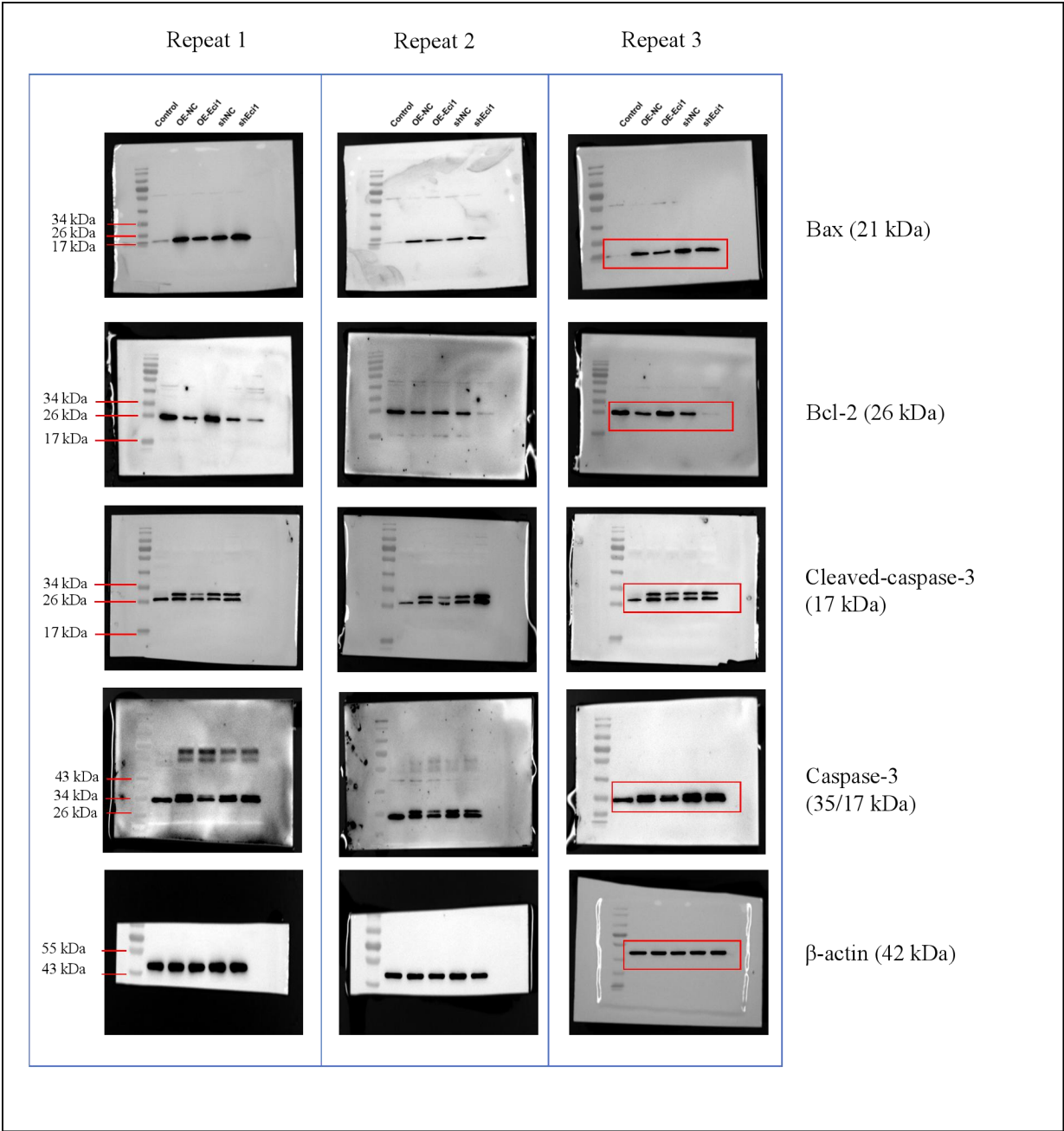

Figure 6E

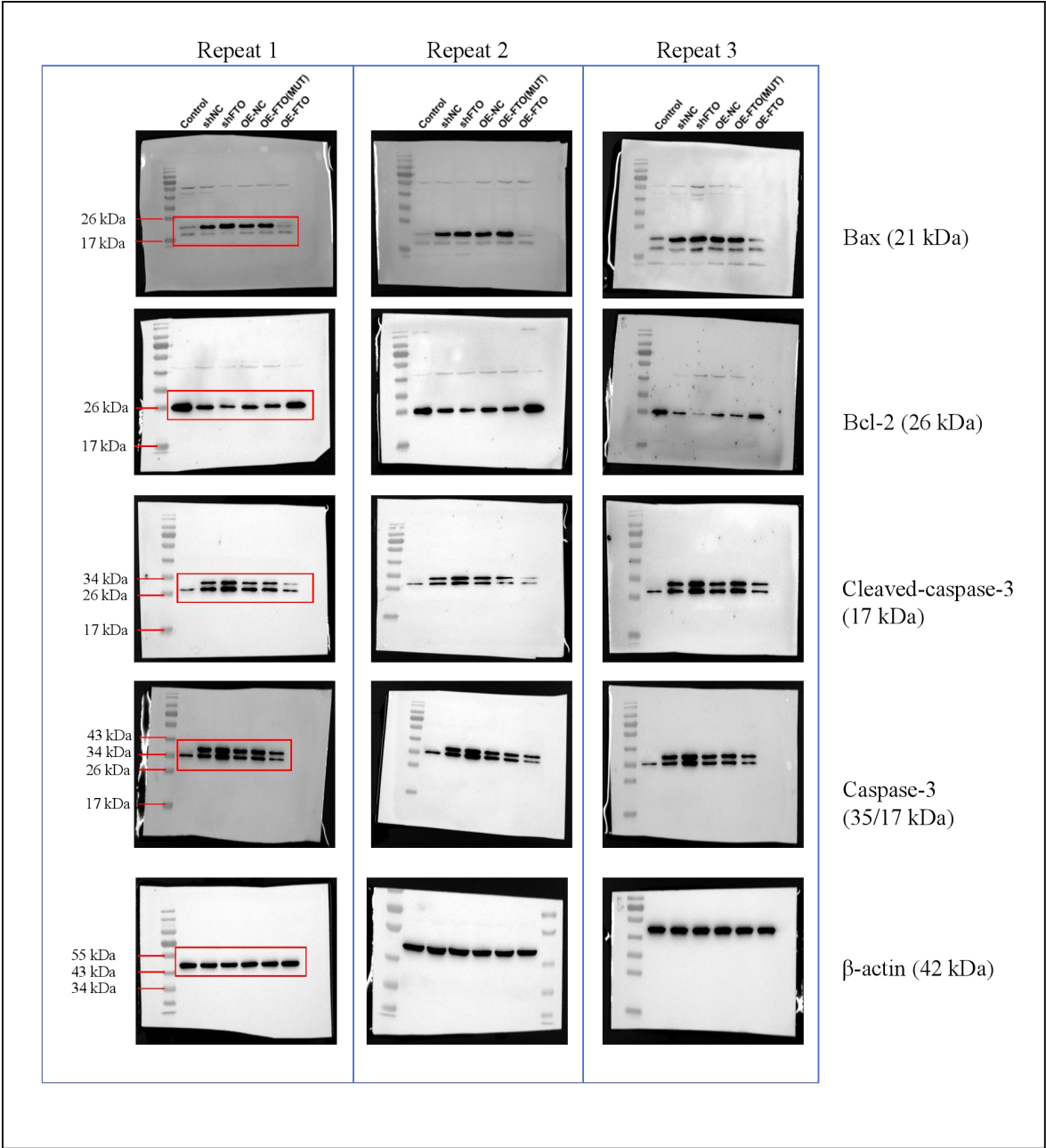

Figure 6F

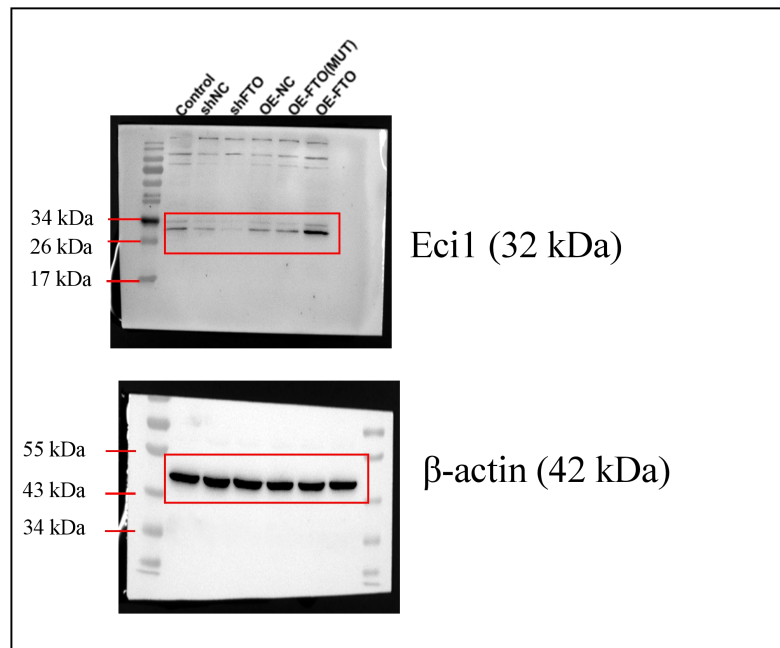

Supplement: Supplementary file 1 [file biomolecules-16-00098-s001.zip › File S1. biomolecules-4027548 original WB.pdf]
